# Contrasting Patterns of Climatic Niche Divergence in *Trebouxia—*A Clade of Lichen-Forming Algae

**DOI:** 10.3389/fmicb.2022.791546

**Published:** 2022-02-15

**Authors:** Matthew P. Nelsen, Steven D. Leavitt, Kathleen Heller, Lucia Muggia, H. Thorsten Lumbsch

**Affiliations:** ^1^The Field Museum, Negaunee Integrative Research Center, Chicago, IL, United States; ^2^Department of Biology, M. L. Bean Life Science Museum, Brigham Young University, Provo, UT, United States; ^3^Biological Sciences Division, University of Chicago, Chicago, IL, United States; ^4^Department of Life Sciences, University of Trieste, Trieste, Italy

**Keywords:** climate, niche, diversification, lichen, photobiont, *Trebouxia*

## Abstract

Lichen associations are overwhelmingly supported by carbon produced by photosynthetic algal symbionts. These algae have diversified to occupy nearly all climates and continents; however, we have a limited understanding of how their climatic niches have evolved through time. Here we extend previous work and ask whether phylogenetic signal in, and the evolution of, climatic niche, varies across climatic variables, phylogenetic scales, and among algal lineages in *Trebouxia—*the most common genus of lichen-forming algae. Our analyses reveal heterogeneous levels of phylogenetic signal across variables, and that contrasting models of evolution underlie the evolution of climatic niche divergence. Together these analyses demonstrate the variable processes responsible for shaping climatic tolerance in *Trebouxia*, and provide a framework within which to better understand potential responses to climate change-associated perturbations. Such predictions reveal a disturbing trend in which the pace at which modern climate change is proceeding will vastly exceed the rate at which *Trebouxia* climatic niches have previously evolved.

## Introduction

Closely related species have a tendency to resemble one another as a consequence of their shared evolutionary history (Darwin, 1859; Blomberg and Garland, 2002). Thus, related lineages may occupy similar ecological niches and overlap in their climatic tolerance. However, the extent to which lineages resemble one another may vary both by trait and across clades as a result of variable evolutionary processes. As climate change has, and is predicted to, disrupt ecosystems and force ranges to shift, it is important to understand which lineages may be especially vulnerable. This vulnerability, is in part, a product of climatic preference. Lineages with slow rates of climatic niche evolution may have a more limited ability to adapt to and persist through climatic perturbations than those with a legacy of evolutionary lability (Holt, 1990; Wiens et al., 2010). Indeed, estimates of predicted climate change often vastly outpace past rates of climatic niche evolution in diverse lineages, suggesting these species may not be able to adapt to the rapid climate change that is underway (Quintero and Wiens, 2013; Jezkova and Wiens, 2016). Thus, it is important to understand how the climatic niche of a lineage has evolved through time in order to identify its potential sensitivity or ability to adapt to shifting climates.

Symbioses are expected to be especially vulnerable to climate change (Dunn et al., 2009; Colwell et al., 2012; Kikuchi et al., 2016; Sampayo et al., 2016; Baker et al., 2018; Rolshausen et al., 2018). Lichens—symbiotic associations between heterotrophic fungi and photosynthetic microbes (eukaryotic microalgae and/or cyanobacteria)—make important and diverse ecosystem contributions (Seaward, 2008; Elbert et al., 2012; Porada et al., 2014, 2018; Rutherford et al., 2017), and are well-known bioindicators (Nimis et al., 2002; Giordani and Incerti, 2008; Will-Wolf et al., 2015; Loppi, 2019; Will-Wolf and Jovan, 2019) with certain lichens predicted to exhibit strong responses to changing climates (Allen and Lendemer, 2016; Ellis, 2019; Nelsen and Lumbsch, 2020). Thus, understanding the evolutionary legacy of climatic niche evolution in these symbionts is paramount to predicting their extinction vulnerability and potential to adapt to changing climates.

The eukaryotic algal genus *Trebouxia* is the most common lichen photobiont (Tschermak-Woess, 1988; Rambold et al., 1998; Voytsekhovich et al., 2011; Miadlikowska et al., 2014; Nelsen et al., 2021). It occurs in nearly all terrestrial ecosystems, but is especially frequent in more temperate-boreal/alpine ecosystems (Honegger, 1998, 2009; Sanders, 2001; Pérez-Ortega et al., 2012; Ruprecht et al., 2012; Wagner et al., 2020; Nelsen et al., 2021). Global efforts to characterize the environmental preferences of *Asterochloris*, a *Trebouxia* relative, have revealed the differential climatic tolerance of individual species-level groups, while suggesting closely related accessions shared similar environmental preferences and exhibited strong phylogenetic conservatism (Peksa and Škaloud, 2011; Vančurová et al., 2018; Kosecka et al., 2021). Previous multivariate efforts in *Trebouxia* have revealed coarse-scaled trends such that members of a specific clade (*Trebouxia* clade “C”) tended to exhibit a more limited climatic preference than others, and a joint analysis of two climatic variables suggested rates of climatic niche diversification across *Trebouxia* have remained constant through time (Nelsen et al., 2021). However, these two variables (mean annual temperature and precipitation) exhibited similar responses in a phylogenetic principal components analysis, and it remains unclear whether preference toward other climatic variables exhibit similar trends and were shaped by similar evolutionary processes. Here we extend previous work (Nelsen et al., 2021) and ask whether climatic niche evolution varies across climatic variables. These past estimates are then used to make predictions about projected responses to climate change.

## Materials and Methods

### General

Here we focused on characterizing phylogenetic signal in, and the evolution of, climatic niche, in the green algal genus *Trebouxia*. While comparable analyses of the over 7,000 fungal species associated with *Trebouxia* algae (Nelsen et al., 2021) would certainly be of interest, this was regarded as beyond the scope of the present study. We extended previous work (Nelsen et al., 2021) by characterizing the phylogenetic signal and evolution of climatic preferences for *Trebouxia* operational taxonomic units (OTUs) across a set of phylogenies, thereby accounting for temporal and topological uncertainty. Briefly, we utilized a previous dataset (Nelsen et al., 2021) that subjected over 6,000 publicly available *Trebouxia* ITS sequences to quality control filters, binned accessions into OTUs, and inferred time-scaled phylogenies using a previously published secondary calibration (Nelsen et al., 2020). Here we utilized 1,001 time-scaled phylogenies (Maximum Clade Credibility [MCC] tree, and a random set of 1,000 trees derived from the posterior) of *Trebouxia* OTUs delimited at a sequence similarity of 97.5% (Nelsen et al., 2021). Geographic coordinates for individual accessions passing quality control filters were previously retrieved from NCBI or obtained from the primary literature and estimates for each of 19 bioclimatic variables (Vega et al., 2017) were extracted for each of the 2,359 accessions representing 81 OTUs (min = 1 accession/OTU, mean = 29.1 accessions/OTU, max = 454 accessions/OTU) (Nelsen et al., 2021). Mean estimates of each bioclimatic variable for individual OTUs were used in analyses below (Nelsen et al., 2021), and sampling error estimated from raw values (described below). We note that these are not experimentally derived climatic tolerances, and the observed distribution of *Trebouxia* accessions may be due to other factors including but not limited to biotic interactions such as competition, and sampling bias. However, in the absence of experimental studies characterizing tolerance of individual OTUs to thermal and precipitation extremes and variability—both in and out of the lichenized state—we regard these as provisional proxies for their climatic preference.

Previous work identified contrasting correlations among climatic variables and principal component (PC) axes (Nelsen et al., 2021). These variables can be binned into six groups (Supplementary Figure 1). Three variable groups exhibited strongly negative loadings along PC1: Group 1 represented annual mean temperature and specific humidity (BIO1, BIO12); Group 2 consisted of variables representing mean temperature and specific humidity during the warmest and most humid periods (BIO5, BIO8, BIO10, BIO13, BIO16, BIO18); Group 3 was comprised of variables representing mean temperature and specific humidity during the coldest and least humid periods (BIO6, BIO9, BIO11, BIO14, BIO17, BIO19). Two sets of variables were characterized by strong negative loadings along PC2: Group 4 was composed of variables representing temperature seasonality and range (BIO4, BIO7) and Group 5 contained one variable related to specific humidity seasonality (BIO15). Group 6 included one variable linked to isothermality (BIO3) and was most strongly associated with PC3. Finally, mean diurnal range of temperature (BIO2) exhibited an equally negative loading along PC2 and PC3. Here we assess whether patterns of phylogenetic signal and trait evolution are consistent among individual variables within each of these groupings, as well as among groupings. Analyses were performed in the R programming environment (R Core Team, 2014) at the Grainger Bioinformatics Center (Field Museum).

### Phylogenetic Signal in Climatic Niche

We first asked to what extent closely related *Trebouxia* OTUs resembled one another in their climatic niches by quantifying phylogenetic signal individually for 19 climatic variables. Phylogenetic signal represents the tendency (pattern) for related species to resemble one another as expected under a model of random drift or Brownian motion (BM) (Blomberg and Garland, 2002). The degree of phylogenetic signal in climatic preference was estimated for each climatic variable by calculating the K statistic (Blomberg et al., 2003) for mean climate values in phytools (Revell, 2012). *K*-values of 0 indicate trait evolution proceeded independently of the phylogeny, while *K*-values of 1 reflect the expectation under pure BM/random drift along the phylogeny. Values greater than 1 indicate that trait values of closely related taxa are more similar than expected under a BM model. Significance tests were then conducted to determine whether *K*-values were significantly different from *K* = 0 through comparison with 1,000 randomizations. We included within-OTU variance estimates, but as sample sizes for many OTUs were equal to one, our OTU-specific sampling error was instead derived by calculating the pooled variance and dividing by the square root of the sample size for each OTU (Garamszegi, 2014; Slater and Friscia, 2019). Analyses were conducted for all bioclimatic variables using the MCC tree and 1,000 trees randomly sampled from the posterior, thereby integrating over topological and temporal uncertainty.

### Climatic Niche Disparity Through Time

We conducted genus-level disparity through time (DTT) analyses on each climatic variable in geiger (Pennell et al., 2014), which allowed us to ascertain whether relative climatic niche disparity deviated substantially from that expected under BM. Disparity was measured using the average squared Euclidean distance, and the morphological disparity index (MDI) estimated, which reflects the difference in relative disparity of the observed clade with that expected under BM. Negative MDI values indicate that subclade disparity is lower than expected under BM, and is a commonly observed pattern in adaptive radiations, such that trait diversification is concentrated disproportionately early in the phylogeny (Harmon et al., 2003). Significance was assessed by estimating the probability of obtaining a negative MDI value, and was achieved by calculating the proportion of instances in which the relative disparity of the observed clade was equal to or exceeded that observed in each of 1,000 datasets simulated under Brownian motion (BM) (Slater et al., 2010). Analyses were again conducted on the MCC tree and 1,000 topologies derived from the posterior distribution.

### Climatic Niche Evolution

While the estimates of phylogenetic signal measured above quantify an observed pattern, they do not provide insight into the underlying evolutionary and non-evolutionary processes giving rise to the observed signal (Blomberg and Garland, 2002; Revell et al., 2008; Münkemüller et al., 2012). For instance, phylogenetic signal may be a consequence of multiple evolutionary processes (Revell et al., 2008). Consequently, we fit three explicit models of trait evolution to narrow the potential underlying evolutionary processes structuring climatic niche evolution. These three models were used to determine whether the rate of trait evolution has remained constant, increased or decreased through time, and included: (1) a BM or random walk model in which trait variance between pairs of taxa is determined by the amount of shared ancestry, and the rate of trait evolution remains constant through time (Felsenstein, 1973); (2) an Ornstein-Uhlenbeck (OU) model, which is essentially a BM model in which traits are pulled toward an adaptive optimum (Butler and King, 2004). However, on an ultrametric tree, this model is also identical to one in which trait evolution exponentially increases through time (Slater et al., 2012; Uyeda et al., 2015); consequently, an OU model should be favored if rates of evolution are increasing. Finally, we fit (3) an early burst (EB) model in which trait evolution decreases exponentially through time (Harmon et al., 2010). Analyses were conducted at the genus-level, and we included within-OTU variance estimates as described above. Models were fit in geiger (Pennell et al., 2014) for each variable on the MCC tree and a random set of 1,000 trees derived from the posterior, and fits assessed using relative weights of Akaike Information Criterion corrected for small samples (AICc_w_).

In addition, we compared diffusion rate (σ^2^) estimates from two pairs of thermal and moisture variables to determine whether tolerance to minima and maxima evolve at different rates (Araújo et al., 2013; Liu et al., 2020). As in previous work (Liu et al., 2020), thermal tolerance was assessed by comparing rates from BIO5 (Max Temperature of Warmest Month) and BIO6 (Min Temperature of Coldest Month), while moisture tolerance was assessed by comparing rates from BIO16 (Specific Humidity Mean of Most Humid Quarter) and BIO17 (Specific Humidity Mean of Least Humid Quarter). Rate estimates were derived from the best-fit models (above) for the MCC tree and 1,000 trees derived from the posterior. Rates for the MCC tree are reported, together with 95% highest posterior density (HPD) values from across the distribution of trees.

Finally, we estimated absolute rates of climatic niche change for three thermal variables (BIO1: Mean Annual Temperature; BIO5: Max Temperature of Warmest Month; BIO6: Min Temperature of Coldest Month). We then compared projected thermal changes over the next 80 years due to global heating (IPCC, 2014) with historic rates of thermal niche evolution in *Trebouxia* algae to assess whether these algae are likely to adapt in place to increasing temperatures, or if they will be required to migrate to suitable climates. Absolute rates of change were calculated using an approach similar to that applied in previous work (Quintero and Wiens, 2013; Jezkova and Wiens, 2016) for the MCC tree and a random set of 1,000 trees derived from the posterior. For each trait, we transformed individual trees using the best-fit model of evolution and parameter estimates (above) in geiger (Pennell et al., 2014), and subsequently reconstructed ancestral states in phytools. We then calculated the absolute difference between the tip state and its immediate ancestor, and divided this by the time separating the two. In addition, minimum and maximum variable values for each OTU were extracted from previous work (Nelsen et al., 2021) and used to calculate absolute rates of change. Rates for the MCC tree are reported, together with 95% highest posterior density (HPD) values from across the distribution of trees. The MCC tree was plotted together with rate estimates in phytools.

## Results

**FIGURE 1:**
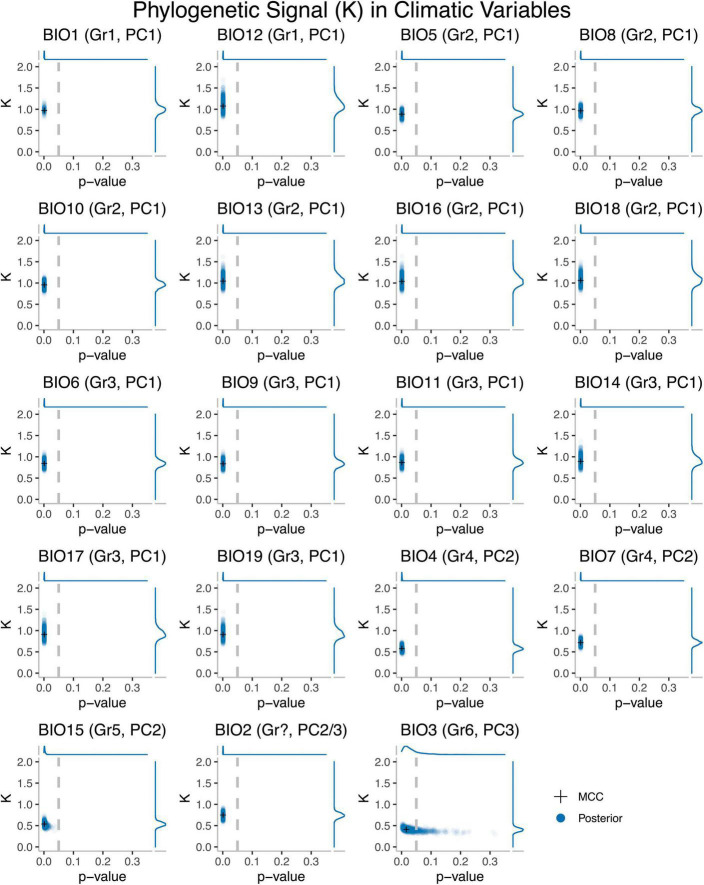
Phylogenetic signal (K) and *p*-values for 19 bioclimatic variables as calculated on the MCC tree (crosses) and 1,000 trees derived from the posterior (circles) for *Trebouxia*. Climatic variable Group 1 includes: Annual Mean Temperature (BIO1), Annual Mean Specific Humidity (BIO12). Climatic variable Group 2 includes: Max Temperature of Warmest Month (BIO5), Mean Temperature of Most Humid Quarter (BIO8), Mean Temperature of Warmest Quarter (BIO10), Specific Humidity of Most Humid Month (BIO13), Specific Humidity Mean of Most Humid Quarter (BIO16), Specific Humidity Mean of Warmest Quarter (BIO18). Climatic variable Group 3 includes: Min Temperature of Coldest Month (BIO6), Mean Temperature of Least Humid Quarter (BIO9), Mean Temperature of Coldest Quarter (BIO11), Specific Humidity of Least Humid Month (BIO14), Specific Humidity Mean of Least Humid Quarter (BIO17), Specific Humidity Mean of Coldest Quarter (BIO19). Climatic variable Group 4 includes: Temperature Seasonality (Standard Deviation * 100) (BIO4), Temperature Annual Range (BIO5–BIO6) (BIO7). Climatic variable Group 5 is limited to Specific Humidity Seasonality (Coefficient of Variation) (BIO15), while Group 6 is restricted to Isothermality (BIO2/BIO7) (*100) (BIO3). It is unclear which group Mean Diurnal Range Temperature (BIO2) belongs to.

We used the K-statistic to evaluate the extent to which climatic preferences of related OTUs resembled one another, relative to those drawn at random. The *K*-values obtained typically ranged from 0.3 to 1 (Figure 1) and were significant in all cases (except for some BIO3 analyses), indicating that climatic preferences were not independent of phylogeny, and those of closely related taxa tended to be more similar to one another than to those of distantly related taxa. Within climatic variable groups, values were broadly consistent. *K*-values for traits from climatic variable Groups 1 (BIO1, BIO12), 2 (BIO5, BIO8, BIO10, BIO13, BIO16, BIO18), and 3 (BIO6, BIO9, BIO11, BIO14, BIO17, BIO19) were all consistent with expectations under BM (*K* = 1) or when trait values for closely related lineages are more similar than expected under a BM model (*K* > 1). In contrast, *K*-values for climatic variable Groups 4 (BIO4, BIO7), 5 (BIO15), 6 (BIO3), and BIO2 were lower than expected under pure BM (*K* = 1).

**FIGURE 2:**
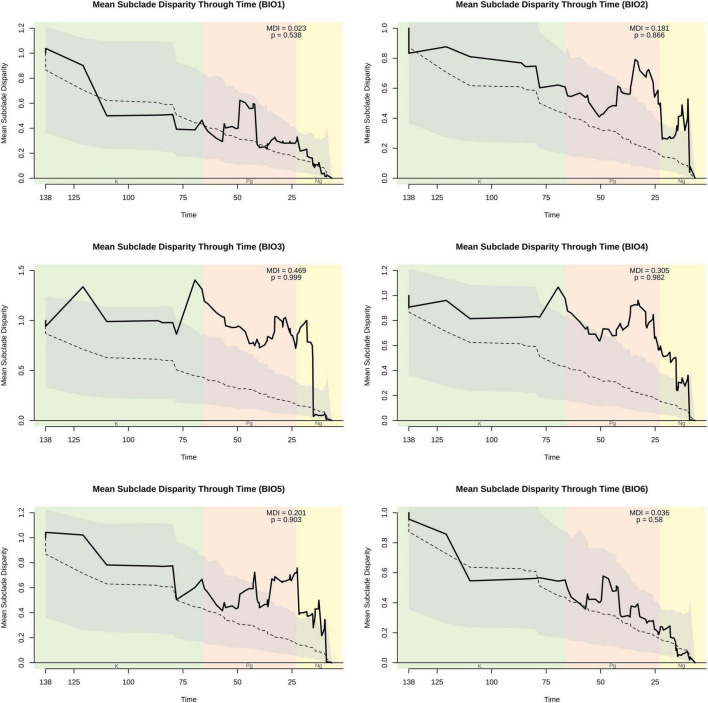
Disparity through time (DTT) plot of BIO1–BIO6 variables. The solid line indicates the observed subclade disparity. The dashed line indicates median estimates from simulated data, and the gray cloud indicates the 95% confidence interval derived from simulated data. The MDI value and its associated *p*-value are provided. Vertical shading indicates geologic periods, which are abbreviated at the bottom. Plots and values are based on analyses of the MCC tree, while a summary of values obtained from the sample of trees derived from the posterior is provided in Supplementary Table 1. DTT plots of BIO7–BIO19 are shown in Supplementary Figures 2, 3.

Our genus-wide disparity through time analyses for individual climatic variables further demonstrated that disparity in these traits was not disproportionately concentrated early in the phylogeny. Mean disparity index (MDI) values for the MCC (Figure 2 and Supplementary Figures 2, 3) and the set of trees derived from the posterior (Supplementary Table 1) were not significantly lower than expected under BM, and therefore, inconsistent with an EB model. In contrast, climatic variable Groups 5 (BIO15), 6 (BIO3), BIO2, and a member of Group 4 (BIO4) regularly yielded MDI values significantly more positive (1—*p*-value, since test is one-sided) than expected under BM, and instead consistent with an OU model of evolution. The MDI values and significance levels achieved for the other variable in climatic variable Group 4 (BIO7) were also consistent with an OU model of evolution, but significance and MDI values were not as extreme as those for BIO4.

**FIGURE 3:**
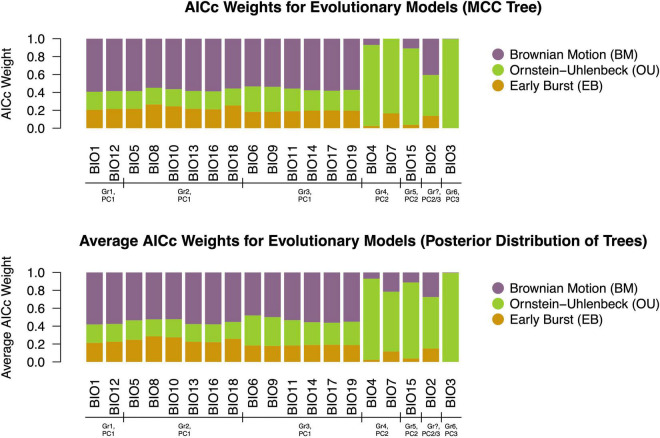
AICc weights for three models fit to 19 bioclimatic variables on the MCC tree and 1,000 trees derived from the posterior. The BM model of evolution best described the evolution of most traits. Exceptions to this trend included BIO3, BIO4, BIO7, and BIO15, in which an OU process was overwhelmingly favored. Additionally, support for an OU model was equal to or slightly higher than that of BM for trait BIO2. For all other traits, support for OU and EB models was comparable, but substantially less than that of BM.

**FIGURE 4:**
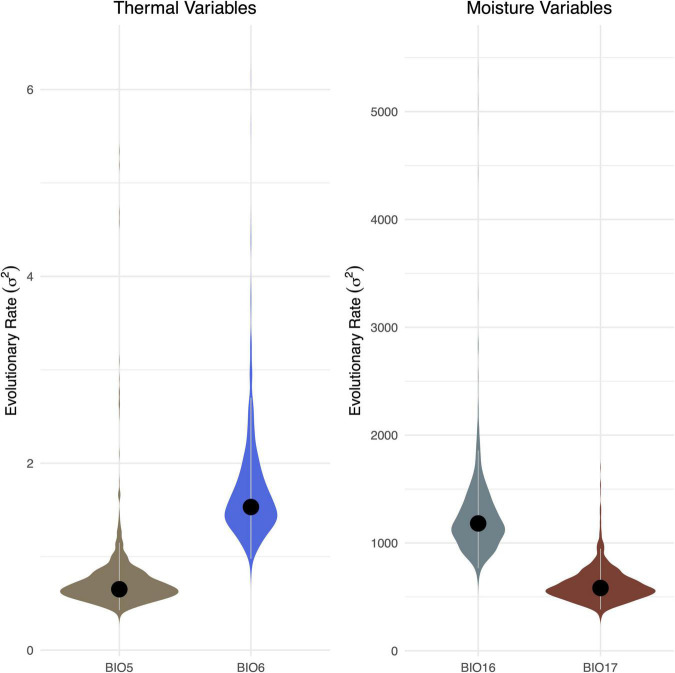
Comparison of rates of evolution among four (BIO5 vs. BIO6; BIO16 vs. BIO17) bioclimatic variables. Values derived from analyses using the MCC tree are indicated with a black dot. Violin plots represent the distribution of values conducted on the 1,000 trees derived from the posterior. The 95% HPD of value is indicated with gray lines.

**FIGURE 5:**
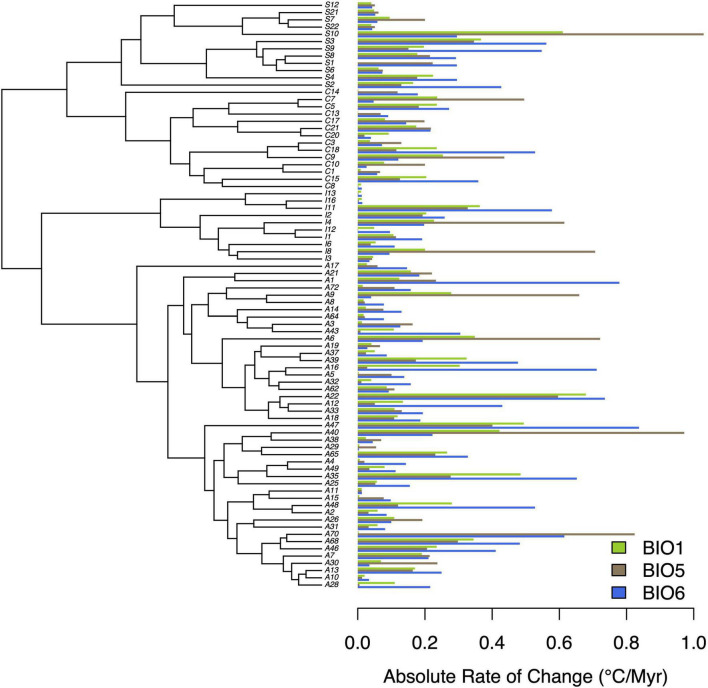
Absolute rates of climatic niche evolution for three thermal variables (BIO1, BIO5, BIO6). Rate estimates are in °C/Myr, and are plotted alongside the MCC tree from previous work (Nelsen et al., 2021).

Our subsequent model-fitting procedure confirmed that a BM model of evolution best characterized the evolution of traits in climatic variable Groups 1 (BIO1, BIO12), 2 (BIO5, BIO8, BIO10, BIO13, BIO16, BIO18), and 3 (BIO6, BIO9, BIO11, BIO14, BIO17, BIO19) (Figure 3). In contrast The OU model was favored for climatic variable Groups 4 (BIO4, BIO7), 5 (BIO15), and 6 (BIO3), while support for an OU model was equal to, or slightly higher than that of BM for climatic variable BIO2. Diffusion rate estimates (σ^2^) for targeted thermal (BIO5, BIO6) and moisture (BIO16, BIO17) variables derived from the best-fit models of evolution (Figure 3) generally suggest that thermal maxima (BIO5) evolved at a slower rate than thermal minima (BIO6), and moisture maxima (BIO16) largely evolved at a faster rate than moisture minima (BIO17) (Figure 4). While there is some overlap among rate estimates within each variable class, distributions appear largely segregated. Finally, our absolute rate estimates of climatic niche evolution varied among OTUs and across variables, but were generally less than 1°C/Myr, regardless of thermal values and approaches employed (Figure 5 and Supplementary Tables 2–4).

## Discussion

Significant phylogenetic signal at the genus level was detected across nearly all variables; however, the magnitude of this signal varied substantially across climatic variables. All variables linked to PC1 (BIO1, BIO12, BIO5, BIO8, BIO10, BIO13, BIO16, BIO18, BIO6, BIO9, BIO11, BIO14, BIO17, BIO19) in previous work (Nelsen et al., 2021) yielded *K*-values consistent with a BM expectation (*K* = 1), DTT plots and MDI values consistent with BM, and had a BM model of evolution overwhelmingly favored in our model-fitting analyses. These climatic variables reflect averages and extreme thermal and specific humidity values, and suggest that at this phylogenetic scale, *Trebouxia* algae have evolved thermal and moisture tolerances under a neutral evolutionary process and at a relatively constant rate.

In contrast, lower (*K* < 1) or non-significant levels of signal were detected at the genus level for a limited number of climatic variables—all (BIO4, BIO7, BIO15, BIO2, BIO3) of which were linked to PC2 and PC3 in previous work (Nelsen et al., 2021), and reflected thermal or moisture variability (seasonally, diurnally). These variables also yielded DTT plots inconsistent with BM, and MDI values consistent with a non-BM process. Moreover, our model-fitting analyses suggested an OU model was almost always overwhelmingly favored for these variables. Together, this may indicate that, at this phylogenetic scale, the evolution of thermal and moisture variability tolerance may be under selection and evolve toward an adaptive optimum. However, it may instead suggest the rate of trait evolution is exponentially increasing through time (AC model, Blomberg et al., 2003), as these two models are equivalent on an ultrametric tree lacking fossil taxa (Slater et al., 2012; Uyeda et al., 2015); thus, we are unable to distinguish among these two patterns. Regardless, it does reveal a departure from Brownian motion and, together with our findings for other climatic variables, indicates variable evolutionary processes underlie climatic niche evolution in this clade of keystone symbionts.

Our DTT plots, MDI values and model-fitting analyses also demonstrate that at this phylogenetic scale, climatic preference under an exponentially decreasing rate of evolution (EB) consistent with that of an adaptive radiation is highly unlikely. Thus, clades have not evolved exceptionally distinct climatic preferences at deep phylogenetic scales, and instead exhibit overlapping climatic tolerances. This is consistent with previous analyses demonstrating the presence of multiple clades in the same geographic locality (Doering and Piercey-Normore, 2009; Werth, 2012; Leavitt et al., 2013, 2015), and observations that climatic niche divergence of annual mean temperature (BIO1) and specific humidity (BIO12) did not jointly evolve under an EB process (Nelsen et al., 2021). Extending our analyses to include numerous individual climatic variables further demonstrates that at the genus level, early bursts in climatic niche tolerance appear rare.

Our analyses of the evolution of thermal and moisture climatic niche limits suggest an asymmetry such that upper thermal limits of *Trebouxia* algae evolve at a slower rate relative to lower thermal limits, and that upper moisture limits evolve at a more rapid rate than lower thermal limits. Both of these findings are consistent with previous work. For instance, a greater range of lower thermal limits relative to upper limits was detected in animals (Araújo et al., 2013), and subsequent work integrating evolutionary the age and relationships of a diverse range of animals and plants has further revealed phylogenetic constraints on thermal limits, with lower limits evolving faster than upper limits (Muñoz et al., 2014; Culumber and Tobler, 2018; Liu et al., 2020; Bennett et al., 2021). We emphasize that these limits may represent hard physiological boundaries that may restrict the potential for lineages to adapt *in situ* to changing climates. Together, these findings suggest the limited potential for several lineages to evolve elevated upper thermal limits in response to increasing global temperatures (Colwell et al., 2008; Liu et al., 2020; Bennett et al., 2021). Similarly, previous work on a range of plants and animals has demonstrated that lower moisture limits evolve at a slower rate than upper limits, suggesting that it may be more difficult to adapt to increased aridity than to increased moisture (Liu et al., 2020). Consistent with other systems, our analyses of the evolutionary legacies of *Trebouxia* algae suggest a limited capacity to adapt *in situ* to increased temperature and decreased moisture (relative to decreased temperature and increased moisture). However, we note that lower thermal limits frequently decrease with increasing latitude, while upper thermal limits remain constant across latitudes (Addo-Bediako et al., 2000); this trend could in turn underlie the observed asymmetric rates of thermal niche evolution, as thermal minima may exhibit greater global variation relative to thermal maxima (Liu et al., 2020). We thus encourage further work to disentangle the roles intrinsic and extrinsic variables have played in structuring these discordant rates of evolution.

Our estimates of absolute rates of climatic niche evolution suggested *Trebouxia* OTUs have evolved at a relatively slow pace, on order with some of the slower rates previously estimated from a diverse range of animals and plants (Quintero and Wiens, 2013; Jezkova and Wiens, 2016). Given that the global heating we are currently experiencing is expected to increase temperatures by 1–4°C (relative to 1986–2005) by the year 2100 (IPCC, 2014)—less than 80 years from now, these algae with their comparatively slow rates of thermal niche evolution [mean rate estimates of 0.13–0.62°C/Myr (BIO1), 0.07–0.71°C/Myr (BIO5), 0.1–1.08°C/Myr (BIO6)] will likely undergo range modification. This suggests that in certain portions of an OTU’s range, individuals are unlikely to adapt *in situ* to increased temperatures experienced at such a rapid rate, and will instead be required to disperse to more habitable locations, thereby altering their distribution. Previous work has emphasized species migration as a means to persist in the face of changing climates; for instance, the Quaternary record illustrates how species ranges have changed in response to shifting climates (Ackerly, 2003). However, this migration is likely to also affect lichen fungi that depend on these algae for nutrition. Such disruptions of biotic interactions are expected to be one of the major drivers of climate change-mediated local extinction (Cahill et al., 2013), with symbioses in particular anticipated to be especially vulnerable to the effects of climate change (Dunn et al., 2009; Colwell et al., 2012; Kikuchi et al., 2016; Sampayo et al., 2016; Baker et al., 2018; Rolshausen et al., 2018). Thus, algal range shifts may further disrupt interaction webs and force fungi to also disperse or acquire new algal symbionts. We emphasize that our climatic niche variables are not direct estimates of the physiological limits of species, but are instead regarded as proxies. Similarly, our characterization of their niches only encompassed their realized niche, rather than their fundamental niche. We therefore stress the need for detailed studies to identify the physiological limits of these algae, both in culture and in the lichenized state. Despite this, our analyses of past rates of climatic niche evolution paint a troubling picture by suggesting a limited evolutionary capacity for *Trebouxia* algae to adapt *in situ* to rapidly increasing temperatures.

## Data Availability Statement

Publicly available datasets were analyzed in this study. Data and code used here are provided at: https://github.com/mpnelsen/Trebouxia_Niche_Divergence.

## Author Contributions

MN, SL, KH, LM, and HL designed research and wrote the manuscript. MN performed research. MN and KH collected, analyzed, and interpreted data. All authors contributed to the article and approved the submitted version.

## Conflict of Interest

The authors declare that the research was conducted in the absence of any commercial or financial relationships that could be construed as a potential conflict of interest.

## Publisher’s Note

All claims expressed in this article are solely those of the authors and do not necessarily represent those of their affiliated organizations, or those of the publisher, the editors and the reviewers. Any product that may be evaluated in this article, or claim that may be made by its manufacturer, is not guaranteed or endorsed by the publisher.

## Supplementary Material

Supplementary Figure 1 The first two axes of the phylogenetic principal component analysis (pPCA) of 19 bioclimatic variables from Nelsen et al. (2021) with OTUs shaded by clade. Axes are labeled with the proportion of variance explained by each axis, and correlations with bioclimatic variables (1–19) are indicated with gray lines. Climatic variables can be binned into six groups with similar correlations to individual axes. Group 1 includes: Annual Mean Temperature (BIO1), Annual Mean Specific Humidity (BIO12). Climatic variable Group 2 includes: Max Temperature of Warmest Month (BIO5), Mean Temperature of Most Humid Quarter (BIO8), Mean Temperature of Warmest Quarter (BIO10), Specific Humidity of Most Humid Month (BIO13), Specific Humidity Mean of Most Humid Quarter (BIO16), Specific Humidity Mean of Warmest Quarter (BIO18). Climatic variable Group 3 includes: Min Temperature of Coldest Month (BIO6), Mean Temperature of Least Humid Quarter (BIO9), Mean Temperature of Coldest Quarter (BIO11), Specific Humidity of Least Humid Month (BIO14), Specific Humidity Mean of Least Humid Quarter (BIO17), Specific Humidity Mean of Coldest Quarter (BIO19). Climatic variable Group 4 includes: Temperature Seasonality (Standard Deviation *100) (BIO4), Temperature Annual Range (BIO5–BIO6) (BIO7). Climatic variable Group 5 is limited to Specific Humidity Seasonality (Coefficient of Variation) (BIO15), while Group 6 is restricted to Isothermality (BIO2/BIO7) (* 100) (BIO3). It is unclear which group Mean Diurnal Range Temperature (BIO2) belongs to. Figure originally published in Nelsen et al. (2021) and reproduced here with permission from Oxford University Press.

Supplementary Figure 2 Disparity through time (DTT) plot of BIO7–BIO12 variables. The solid line indicates the observed subclade disparity. The dashed line indicates median estimates from simulated data, and the gray cloud indicates the 95% confidence interval derived from simulated data. The MDI value and its associated *p*-value are provided. Vertical shading indicates geologic periods, which are abbreviated at the bottom.

Supplementary Figure 3 Disparity through time (DTT) plot of BIO13–BIO19 variables. The solid line indicates the observed subclade disparity. The dashed line indicates median estimates from simulated data, and the gray cloud indicates the 95% confidence interval derived from simulated data. The MDI value and its associated *p*-value are provided. Vertical shading indicates geologic periods, which are abbreviated at the bottom.
