# Supplementary figures and images for: Contrasting Patterns of Climatic Niche Divergence in Trebouxia—A Clade of Lichen-Forming Algae
Source: Front Microbiol. 2022 Feb 15;13:791546. doi: 10.3389/fmicb.2022.791546 (PMC8886231; doi:10.3389/fmicb.2022.791546)

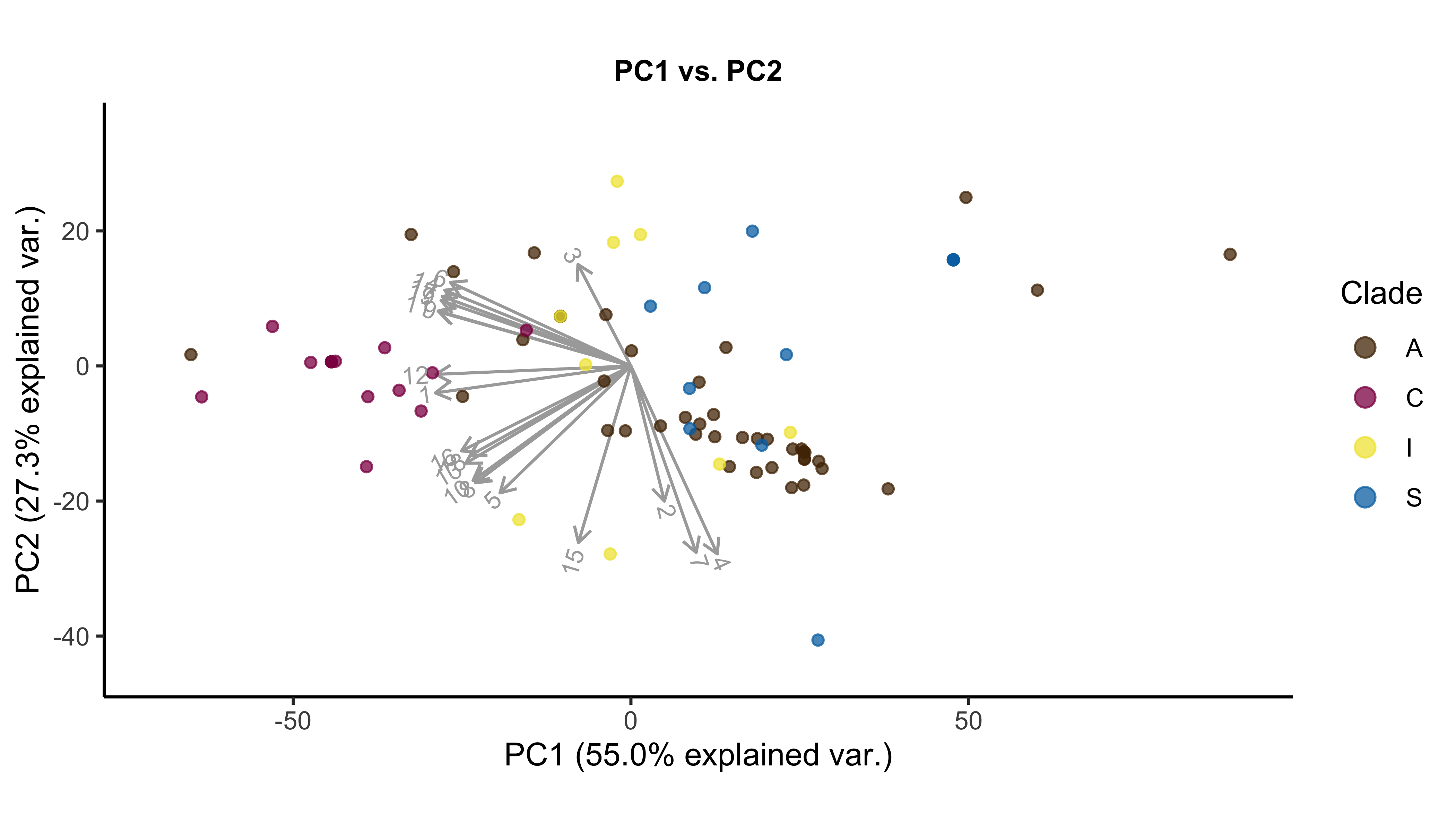

Supplement: Supplementary Figure 1 — The first two axes of the phylogenetic principal component analysis (pPCA) of 19 bioclimatic variables from Nelsen et al. (2021) with OTUs shaded by clade. Axes are labeled with the proportion of variance explained by each axis, and correlations with bioclimatic variables (1–19) are indicated with gray lines. Climatic variables can be binned into six groups with similar correlations to individual axes. Group 1 includes: Annual Mean Temperature (BIO1), Annual Mean Specific Humidity (BIO12). Climatic variable Group 2 includes: Max Temperature of Warmest Month (BIO5), Mean Temperature of Most Humid Quarter (BIO8), Mean Temperature of Warmest Quarter (BIO10), Specific Humidity of Most Humid Month (BIO13), Specific Humidity Mean of Most Humid Quarter (BIO16), Specific Humidity Mean of Warmest Quarter (BIO18). Climatic variable Group 3 includes: Min Temperature of Coldest Month (BIO6), Mean Temperature of Least Humid Quarter (BIO9), Mean Temperature of Coldest Quarter (BIO11), Specific Humidity of Least Humid Month (BIO14), Specific Humidity Mean of Least Humid Quarter (BIO17), Specific Humidity Mean of Coldest Quarter (BIO19). Climatic variable Group 4 includes: Temperature Seasonality (Standard Deviation *100) (BIO4), Temperature Annual Range (BIO5–BIO6) (BIO7). Climatic variable Group 5 is limited to Specific Humidity Seasonality (Coefficient of Variation) (BIO15), while Group 6 is restricted to Isothermality (BIO2/BIO7) (* 100) (BIO3). It is unclear which group Mean Diurnal Range Temperature (BIO2) belongs to. Figure originally published in Nelsen et al. (2021) and reproduced here with permission from Oxford University Press. [file Image_1.JPEG]
