# Supplementary material for: Contrasting Patterns of Climatic Niche Divergence in Trebouxia—A Clade of Lichen-Forming Algae
Source: Front Microbiol. 2022 Feb 15;13:791546. doi: 10.3389/fmicb.2022.791546 (PMC8886231; doi:10.3389/fmicb.2022.791546)
Supplement: Supplementary file 4 [file Table_1.docx]

**Table S1** Summary of DTT analyses conducted on a random set of 1000 trees derived from the posterior distribution. Mean disparity index (MDI) values for the MCC (Figures S2-S3) and the set of trees derived from the posterior were not significantly lower (more negative) than expected under BM, but occasionally higher (more positive) than expected under BM, and consistent with an OU model of evolution (p-value of 0.975 or greater).

|  | **MDI (Min)** | **MDI (Mean)** | **MDI (Max)** | **P-value (Min)** | **P-value (Mean)** | **P-value (Max)** |
| --- | --- | --- | --- | --- | --- | --- |
| BIO1 | -0.14 | 0.0029 | 0.239 | 0.12 | 0.4775 | 0.939 |
| BIO2 | 0.071 | 0.1899 | 0.308 | 0.641 | 0.8743 | 0.983 |
| BIO3 | 0.199 | 0.4847 | 0.984 | 0.841 | 0.9974 | 1 |
| BIO4 | 0.112 | 0.3196 | 0.516 | 0.725 | 0.9761 | 1 |
| BIO5 | -0.007 | 0.1879 | 0.368 | 0.45 | 0.8632 | 0.998 |
| BIO6 | -0.099 | 0.0362 | 0.202 | 0.21 | 0.5627 | 0.886 |
| BIO7 | 0.077 | 0.24 | 0.369 | 0.66 | 0.9304 | 0.993 |
| BIO8 | -0.067 | 0.1185 | 0.333 | 0.312 | 0.7454 | 0.981 |
| BIO9 | -0.12 | 0.0049 | 0.203 | 0.173 | 0.4834 | 0.906 |
| BIO10 | -0.061 | 0.121 | 0.321 | 0.323 | 0.7507 | 0.986 |
| BIO11 | -0.126 | -0.0025 | 0.199 | 0.14 | 0.4642 | 0.882 |
| BIO12 | -0.184 | -0.0736 | 0.087 | 0.048 | 0.2902 | 0.664 |
| BIO13 | -0.124 | 0.0173 | 0.173 | 0.146 | 0.5137 | 0.834 |
| BIO14 | -0.168 | -0.0355 | 0.115 | 0.086 | 0.382 | 0.762 |
| BIO15 | 0.244 | 0.502 | 0.812 | 0.908 | 0.9987 | 1 |
| BIO16 | -0.144 | -0.0039 | 0.159 | 0.127 | 0.4594 | 0.807 |
| BIO17 | -0.17 | -0.0437 | 0.11 | 0.083 | 0.3624 | 0.764 |
| BIO18 | -0.13 | 0.0055 | 0.179 | 0.139 | 0.4826 | 0.831 |
| BIO19 | -0.143 | -0.0355 | 0.115 | 0.109 | 0.3818 | 0.75 |
