# Supplementary material for: Contrasting Patterns of Climatic Niche Divergence in Trebouxia—A Clade of Lichen-Forming Algae
Source: Front Microbiol. 2022 Feb 15;13:791546. doi: 10.3389/fmicb.2022.791546 (PMC8886231; doi:10.3389/fmicb.2022.791546)
Supplement: Supplementary file 5 [file Table_2.docx]

**Table S2** Summary data for absolute rates of climatic niche evolution for BIO1. OTU numbers are listed together with the modern mean value (ModMeanVal), the modern minimum value (ModMinVal), the modern maximum value (ModMaxVal), stem age from the MCC tree (Age), ancestral value based on the MCC tree (AncVal), the absolute rate of change using values from the MCC tree and the modern mean value (MCCAbsRateMean), the absolute rate of change using values from the MCC tree and the modern minimum value (MCCAbsRateMin), and the absolute rate of change using values from the MCC tree and the modern maximum value (MCCAbsRateMax). Rate estimates were also calculated across a distribution of 1000 trees derived from the posterior, and mean values presented, together with upper and lower limits of the 95% HPD. These include the: mean (MeanAbsRate_Mean), lower 95% HPD (MeanLower95Rate) and upper 95% HPD (MeanUpper95Rate) estimated rate of change using the modern mean value; mean (MinAbsRate_Mean), lower 95% HPD (MinLower95Rate) and upper 95% HPD (MinUpper95Rate) estimated rate of change using the modern minimum value; mean (MaxAbsRate_Mean), lower 95% HPD (MaxLower95Rate) and upper 95% HPD (MaxUpper95Rate) estimated rate of change using the modern maximum value.

|  | ModMeanVal | ModMinVal | ModMaxVal | Age | AncVal | MCCAbsRateMean | MCCAbsRateMin | MCCAbsRateMax | MeanAbsRate_Mean | MeanLower95Rate | MeanUpper95Rate | MinAbsRate_Mean | MinLower95Rate | MinUpper95Rate | MaxAbsRate_Mean | MaxLower95Rate | MaxUpper95Rate |
| --- | --- | --- | --- | --- | --- | --- | --- | --- | --- | --- | --- | --- | --- | --- | --- | --- | --- |
| A_97.5_1 | -9.54 | -33.50 | 14.13 | 37.54 | -0.70 | 0.24 | 0.87 | 0.40 | 0.27 | 0.15 | 0.41 | 0.95 | 0.43 | 1.67 | 0.41 | 0.07 | 0.92 |
| A_97.5_2 | 7.55 | -3.40 | 18.37 | 12.70 | 7.51 | 0.00 | 0.86 | 0.85 | 0.02 | 0.00 | 0.05 | 1.05 | 0.31 | 2.18 | 1.05 | 0.30 | 2.24 |
| A_97.5_3 | 13.34 | -5.57 | 24.43 | 21.25 | 15.30 | 0.09 | 0.98 | 0.43 | 0.11 | 0.00 | 0.26 | 1.05 | 0.40 | 1.86 | 0.45 | 0.24 | 0.74 |
| A_97.5_4 | 7.42 | -4.27 | 21.63 | 14.70 | 4.43 | 0.20 | 0.59 | 1.17 | 0.23 | 0.04 | 0.52 | 0.62 | 0.23 | 1.26 | 1.26 | 0.40 | 2.66 |
| A_97.5_5 | 9.32 | -4.27 | 13.30 | 15.49 | 14.95 | 0.36 | 1.24 | 0.11 | 0.36 | 0.12 | 0.69 | 1.24 | 0.49 | 2.36 | 0.10 | 0.01 | 0.22 |
| A_97.5_6 | 12.77 | -23.87 | 23.33 | 42.97 | 11.61 | 0.03 | 0.83 | 0.27 | 0.03 | 0.00 | 0.07 | 0.90 | 0.44 | 1.47 | 0.28 | 0.17 | 0.44 |
| A_97.5_7 | 6.63 | -0.03 | 14.40 | 16.54 | 7.47 | 0.05 | 0.45 | 0.42 | 0.04 | 0.01 | 0.08 | 0.55 | 0.21 | 0.98 | 0.55 | 0.18 | 1.04 |
| A_97.5_8 | 17.39 | 17.03 | 18.43 | 23.78 | 9.68 | 0.32 | 0.31 | 0.37 | 0.35 | 0.17 | 0.61 | 0.33 | 0.15 | 0.57 | 0.40 | 0.20 | 0.68 |
| A_97.5_9 | 9.66 | -6.63 | 22.43 | 23.78 | 9.68 | 0.00 | 0.69 | 0.54 | 0.05 | 0.00 | 0.15 | 0.73 | 0.30 | 1.29 | 0.58 | 0.27 | 0.95 |
| A_97.5_10 | 10.58 | 1.27 | 22.43 | 6.70 | 8.70 | 0.28 | 1.11 | 2.05 | 0.30 | 0.13 | 0.53 | 1.20 | 0.35 | 2.34 | 2.22 | 0.83 | 4.27 |
| A_97.5_11 | 9.86 | -0.03 | 17.30 | 22.68 | 8.65 | 0.05 | 0.38 | 0.38 | 0.06 | 0.02 | 0.10 | 0.39 | 0.15 | 0.75 | 0.40 | 0.17 | 0.71 |
| A_97.5_12 | 22.21 | 20.63 | 25.37 | 14.40 | 17.25 | 0.34 | 0.24 | 0.56 | 0.40 | 0.16 | 0.69 | 0.27 | 0.09 | 0.49 | 0.64 | 0.26 | 1.13 |
| A_97.5_13 | 8.43 | 1.27 | 19.57 | 6.70 | 8.70 | 0.04 | 1.11 | 1.62 | 0.05 | 0.00 | 0.16 | 1.20 | 0.37 | 2.34 | 1.76 | 0.67 | 3.39 |
| A_97.5_14 | 14.67 | 6.27 | 23.53 | 36.44 | 10.35 | 0.12 | 0.11 | 0.36 | 0.12 | 0.02 | 0.23 | 0.16 | 0.03 | 0.33 | 0.41 | 0.21 | 0.68 |
| A_97.5_15 | 8.85 | 1.43 | 18.37 | 22.68 | 8.65 | 0.01 | 0.32 | 0.43 | 0.02 | 0.00 | 0.04 | 0.33 | 0.13 | 0.63 | 0.46 | 0.18 | 0.81 |
| A_97.5_16 | 20.63 | 20.63 | 20.63 | 15.49 | 14.95 | 0.37 | 0.37 | 0.37 | 0.37 | 0.17 | 0.71 | 0.37 | 0.17 | 0.71 | 0.37 | 0.17 | 0.71 |
| A_97.5_17 | 13.80 | 13.80 | 13.80 | 79.97 | 8.85 | 0.06 | 0.06 | 0.06 | 0.07 | 0.03 | 0.12 | 0.07 | 0.03 | 0.12 | 0.07 | 0.03 | 0.12 |
| A_97.5_18 | 13.43 | 12.17 | 14.70 | 22.96 | 14.74 | 0.06 | 0.11 | 0.00 | 0.13 | 0.01 | 0.42 | 0.20 | 0.04 | 0.57 | 0.06 | 0.00 | 0.27 |
| A_97.5_19 | 20.63 | 20.63 | 20.63 | 26.18 | 16.50 | 0.16 | 0.16 | 0.16 | 0.20 | 0.10 | 0.32 | 0.20 | 0.10 | 0.32 | 0.20 | 0.10 | 0.32 |
| A_97.5_21 | -4.27 | -4.27 | -4.27 | 37.54 | -0.70 | 0.09 | 0.09 | 0.09 | 0.12 | 0.00 | 0.22 | 0.12 | 0.00 | 0.22 | 0.12 | 0.00 | 0.22 |
| A_97.5_22 | 6.27 | 6.27 | 6.27 | 18.00 | 15.16 | 0.49 | 0.49 | 0.49 | 0.54 | 0.23 | 0.89 | 0.54 | 0.23 | 0.89 | 0.54 | 0.23 | 0.89 |
| A_97.5_25 | -4.27 | -4.27 | -4.27 | 36.11 | 4.89 | 0.25 | 0.25 | 0.25 | 0.28 | 0.16 | 0.44 | 0.28 | 0.16 | 0.44 | 0.28 | 0.16 | 0.44 |
| A_97.5_26 | 12.73 | 7.10 | 18.37 | 29.31 | 10.43 | 0.08 | 0.11 | 0.27 | 0.09 | 0.03 | 0.15 | 0.11 | 0.02 | 0.24 | 0.29 | 0.16 | 0.44 |
| A_97.5_28 | 6.84 | 6.80 | 6.93 | 9.90 | 7.94 | 0.11 | 0.11 | 0.10 | 0.13 | 0.04 | 0.25 | 0.13 | 0.04 | 0.26 | 0.12 | 0.03 | 0.24 |
| A_97.5_29 | 7.90 | 7.07 | 10.40 | 25.95 | 7.58 | 0.01 | 0.02 | 0.11 | 0.03 | 0.00 | 0.08 | 0.05 | 0.00 | 0.13 | 0.09 | 0.02 | 0.16 |
| A_97.5_30 | 6.16 | 3.50 | 10.63 | 12.92 | 7.55 | 0.11 | 0.31 | 0.24 | 0.10 | 0.04 | 0.17 | 0.34 | 0.15 | 0.58 | 0.30 | 0.11 | 0.54 |
| A_97.5_31 | 15.27 | 15.27 | 15.27 | 29.31 | 10.43 | 0.16 | 0.16 | 0.16 | 0.18 | 0.10 | 0.27 | 0.18 | 0.10 | 0.27 | 0.18 | 0.10 | 0.27 |
| A_97.5_32 | 11.35 | 2.07 | 20.63 | 18.46 | 14.49 | 0.17 | 0.67 | 0.33 | 0.17 | 0.04 | 0.32 | 0.66 | 0.23 | 1.14 | 0.32 | 0.14 | 0.55 |
| A_97.5_33 | 20.63 | 20.63 | 20.63 | 14.40 | 17.25 | 0.24 | 0.24 | 0.24 | 0.26 | 0.09 | 0.47 | 0.26 | 0.09 | 0.47 | 0.26 | 0.09 | 0.47 |
| A_97.5_35 | 8.50 | 8.50 | 8.50 | 23.67 | 5.32 | 0.13 | 0.13 | 0.13 | 0.14 | 0.05 | 0.27 | 0.14 | 0.05 | 0.27 | 0.14 | 0.05 | 0.27 |
| A_97.5_37 | 21.70 | 21.70 | 21.70 | 9.21 | 19.86 | 0.20 | 0.20 | 0.20 | 0.23 | 0.11 | 0.39 | 0.23 | 0.11 | 0.39 | 0.23 | 0.11 | 0.39 |
| A_97.5_38 | -3.53 | -3.53 | -3.53 | 21.81 | 5.67 | 0.42 | 0.42 | 0.42 | 0.46 | 0.20 | 0.78 | 0.46 | 0.20 | 0.78 | 0.46 | 0.20 | 0.78 |
| A_97.5_39 | 19.83 | 19.83 | 19.83 | 9.21 | 19.86 | 0.00 | 0.00 | 0.00 | 0.05 | 0.00 | 0.13 | 0.05 | 0.00 | 0.13 | 0.05 | 0.00 | 0.13 |
| A_97.5_40 | 13.27 | 13.27 | 13.27 | 21.81 | 5.67 | 0.35 | 0.35 | 0.35 | 0.39 | 0.15 | 0.71 | 0.39 | 0.15 | 0.71 | 0.39 | 0.15 | 0.71 |
| A_97.5_43 | 25.60 | 25.60 | 25.60 | 21.25 | 15.30 | 0.48 | 0.48 | 0.48 | 0.51 | 0.27 | 0.83 | 0.51 | 0.27 | 0.83 | 0.51 | 0.27 | 0.83 |
| A_97.5_46 | 6.93 | 6.93 | 6.93 | 17.78 | 7.29 | 0.02 | 0.02 | 0.02 | 0.02 | 0.01 | 0.04 | 0.02 | 0.01 | 0.04 | 0.02 | 0.01 | 0.04 |
| A_97.5_47 | 5.73 | 5.73 | 5.73 | 50.58 | 6.96 | 0.02 | 0.02 | 0.02 | 0.02 | 0.00 | 0.04 | 0.02 | 0.00 | 0.04 | 0.02 | 0.00 | 0.04 |
| A_97.5_48 | 6.93 | 6.93 | 6.93 | 12.70 | 7.51 | 0.05 | 0.05 | 0.05 | 0.05 | 0.02 | 0.10 | 0.05 | 0.02 | 0.10 | 0.05 | 0.02 | 0.10 |
| A_97.5_49 | -0.03 | -0.03 | -0.03 | 14.70 | 4.43 | 0.30 | 0.30 | 0.30 | 0.32 | 0.11 | 0.61 | 0.32 | 0.11 | 0.61 | 0.32 | 0.11 | 0.61 |
| A_97.5_62 | 15.77 | 15.77 | 15.77 | 18.46 | 14.49 | 0.07 | 0.07 | 0.07 | 0.05 | 0.00 | 0.12 | 0.05 | 0.00 | 0.12 | 0.05 | 0.00 | 0.12 |
| A_97.5_64 | 3.47 | 3.47 | 3.47 | 32.14 | 11.02 | 0.24 | 0.24 | 0.24 | 0.22 | 0.08 | 0.42 | 0.22 | 0.08 | 0.42 | 0.22 | 0.08 | 0.42 |
| A_97.5_65 | 10.33 | 10.33 | 10.33 | 25.95 | 7.58 | 0.11 | 0.11 | 0.11 | 0.09 | 0.02 | 0.16 | 0.09 | 0.02 | 0.16 | 0.09 | 0.02 | 0.16 |
| A_97.5_68 | 6.93 | 6.93 | 6.93 | 15.06 | 7.20 | 0.02 | 0.02 | 0.02 | 0.02 | 0.01 | 0.04 | 0.02 | 0.01 | 0.04 | 0.02 | 0.01 | 0.04 |
| A_97.5_70 | 6.93 | 6.93 | 6.93 | 15.06 | 7.20 | 0.02 | 0.02 | 0.02 | 0.02 | 0.01 | 0.04 | 0.02 | 0.01 | 0.04 | 0.02 | 0.01 | 0.04 |
| A_97.5_72 | -23.87 | -23.87 | -23.87 | 41.15 | 4.07 | 0.68 | 0.68 | 0.68 | 0.71 | 0.37 | 1.13 | 0.71 | 0.37 | 1.13 | 0.71 | 0.37 | 1.13 |
| I_97.5_1 | 5.85 | -12.80 | 24.47 | 11.39 | 2.68 | 0.28 | 1.36 | 1.91 | 0.32 | 0.01 | 0.79 | 1.56 | 0.57 | 2.85 | 2.20 | 0.63 | 4.42 |
| I_97.5_2 | 12.93 | 12.93 | 12.93 | 52.57 | 12.17 | 0.01 | 0.01 | 0.01 | 0.02 | 0.00 | 0.08 | 0.02 | 0.00 | 0.08 | 0.02 | 0.00 | 0.08 |
| I_97.5_3 | 18.27 | 15.90 | 20.63 | 36.38 | 14.31 | 0.11 | 0.04 | 0.17 | 0.11 | 0.05 | 0.20 | 0.05 | 0.00 | 0.11 | 0.18 | 0.09 | 0.29 |
| I_97.5_4 | 9.17 | 1.87 | 14.13 | 24.85 | 7.15 | 0.08 | 0.21 | 0.28 | 0.09 | 0.00 | 0.23 | 0.23 | 0.12 | 0.35 | 0.30 | 0.09 | 0.59 |
| I_97.5_6 | 11.81 | 7.37 | 20.63 | 40.05 | 13.27 | 0.04 | 0.15 | 0.18 | 0.02 | 0.00 | 0.06 | 0.12 | 0.06 | 0.21 | 0.23 | 0.10 | 0.37 |
| I_97.5_8 | 20.63 | 20.63 | 20.63 | 36.38 | 14.31 | 0.17 | 0.17 | 0.17 | 0.17 | 0.09 | 0.28 | 0.17 | 0.09 | 0.28 | 0.17 | 0.09 | 0.28 |
| I_97.5_11 | 9.00 | 9.00 | 9.00 | 15.25 | 12.46 | 0.23 | 0.23 | 0.23 | 0.23 | 0.07 | 0.45 | 0.23 | 0.07 | 0.45 | 0.23 | 0.07 | 0.45 |
| I_97.5_12 | -4.27 | -4.27 | -4.27 | 11.39 | 2.68 | 0.61 | 0.61 | 0.61 | 0.70 | 0.31 | 1.21 | 0.70 | 0.31 | 1.21 | 0.70 | 0.31 | 1.21 |
| I_97.5_13 | 12.23 | 12.23 | 12.23 | 32.97 | 12.29 | 0.00 | 0.00 | 0.00 | 0.01 | 0.00 | 0.01 | 0.01 | 0.00 | 0.01 | 0.01 | 0.00 | 0.01 |
| I_97.5_16 | 16.07 | 16.07 | 16.07 | 15.25 | 12.46 | 0.24 | 0.24 | 0.24 | 0.24 | 0.08 | 0.46 | 0.24 | 0.08 | 0.46 | 0.24 | 0.08 | 0.46 |
| C_97.5_1 | 21.40 | 18.63 | 23.97 | 16.69 | 24.58 | 0.19 | 0.36 | 0.04 | 0.22 | 0.05 | 0.52 | 0.42 | 0.10 | 0.92 | 0.05 | 0.00 | 0.14 |
| C_97.5_3 | 18.43 | 11.80 | 24.27 | 9.98 | 20.66 | 0.22 | 0.89 | 0.36 | 0.27 | 0.07 | 0.56 | 1.06 | 0.27 | 2.21 | 0.43 | 0.10 | 0.90 |
| C_97.5_5 | 22.01 | 18.63 | 24.40 | 10.16 | 21.41 | 0.06 | 0.27 | 0.29 | 0.07 | 0.01 | 0.15 | 0.32 | 0.09 | 0.67 | 0.34 | 0.08 | 0.72 |
| C_97.5_7 | 20.60 | 18.63 | 22.57 | 10.16 | 21.41 | 0.08 | 0.27 | 0.11 | 0.09 | 0.02 | 0.19 | 0.32 | 0.09 | 0.67 | 0.13 | 0.03 | 0.28 |
| C_97.5_8 | 21.98 | 20.13 | 24.70 | 42.94 | 22.17 | 0.00 | 0.05 | 0.06 | 0.01 | 0.00 | 0.02 | 0.05 | 0.02 | 0.10 | 0.06 | 0.03 | 0.10 |
| C_97.5_9 | 21.08 | 20.43 | 21.77 | 23.38 | 20.94 | 0.01 | 0.02 | 0.04 | 0.01 | 0.00 | 0.02 | 0.02 | 0.00 | 0.04 | 0.04 | 0.01 | 0.08 |
| C_97.5_10 | 29.02 | 28.67 | 29.37 | 16.69 | 24.58 | 0.27 | 0.24 | 0.29 | 0.30 | 0.12 | 0.60 | 0.28 | 0.11 | 0.55 | 0.33 | 0.13 | 0.66 |
| C_97.5_13 | 24.40 | 24.40 | 24.40 | 40.15 | 22.03 | 0.06 | 0.06 | 0.06 | 0.06 | 0.03 | 0.09 | 0.06 | 0.03 | 0.09 | 0.06 | 0.03 | 0.09 |
| C_97.5_14 | 21.97 | 21.97 | 21.97 | 84.80 | 17.83 | 0.05 | 0.05 | 0.05 | 0.04 | 0.01 | 0.07 | 0.04 | 0.01 | 0.07 | 0.04 | 0.01 | 0.07 |
| C_97.5_15 | 23.22 | 18.73 | 27.70 | 42.94 | 22.17 | 0.02 | 0.08 | 0.13 | 0.02 | 0.01 | 0.04 | 0.09 | 0.04 | 0.15 | 0.14 | 0.08 | 0.22 |
| C_97.5_17 | 22.50 | 22.50 | 22.50 | 36.31 | 22.06 | 0.01 | 0.01 | 0.01 | 0.01 | 0.00 | 0.02 | 0.01 | 0.00 | 0.02 | 0.01 | 0.00 | 0.02 |
| C_97.5_18 | 22.70 | 22.70 | 22.70 | 9.98 | 20.66 | 0.20 | 0.20 | 0.20 | 0.24 | 0.06 | 0.51 | 0.24 | 0.06 | 0.51 | 0.24 | 0.06 | 0.51 |
| C_97.5_20 | 22.70 | 22.70 | 22.70 | 9.00 | 22.61 | 0.01 | 0.01 | 0.01 | 0.01 | 0.00 | 0.02 | 0.01 | 0.00 | 0.02 | 0.01 | 0.00 | 0.02 |
| C_97.5_21 | 22.70 | 22.70 | 22.70 | 9.00 | 22.61 | 0.01 | 0.01 | 0.01 | 0.01 | 0.00 | 0.02 | 0.01 | 0.00 | 0.02 | 0.01 | 0.00 | 0.02 |
| S_97.5_1 | 5.30 | -23.53 | 24.07 | 15.72 | 8.09 | 0.18 | 2.01 | 1.02 | 0.17 | 0.08 | 0.27 | 2.14 | 1.14 | 3.57 | 1.11 | 0.58 | 1.86 |
| S_97.5_2 | 8.54 | -5.63 | 22.20 | 86.68 | 8.92 | 0.00 | 0.17 | 0.15 | 0.01 | 0.00 | 0.03 | 0.19 | 0.11 | 0.29 | 0.19 | 0.08 | 0.31 |
| S_97.5_3 | 11.26 | 2.50 | 14.20 | 28.08 | 8.19 | 0.11 | 0.20 | 0.21 | 0.12 | 0.05 | 0.22 | 0.23 | 0.10 | 0.39 | 0.24 | 0.10 | 0.43 |
| S_97.5_4 | 8.66 | 2.10 | 24.93 | 49.85 | 8.07 | 0.01 | 0.12 | 0.34 | 0.01 | 0.00 | 0.03 | 0.13 | 0.06 | 0.19 | 0.35 | 0.19 | 0.53 |
| S_97.5_6 | 8.02 | 7.80 | 8.23 | 19.53 | 8.01 | 0.00 | 0.01 | 0.01 | 0.02 | 0.00 | 0.05 | 0.01 | 0.00 | 0.04 | 0.03 | 0.00 | 0.07 |
| S_97.5_7 | -4.27 | -4.27 | -4.27 | 14.43 | -3.69 | 0.04 | 0.04 | 0.04 | 0.04 | 0.02 | 0.07 | 0.04 | 0.02 | 0.07 | 0.04 | 0.02 | 0.07 |
| S_97.5_8 | 11.18 | 9.47 | 12.90 | 15.72 | 8.09 | 0.20 | 0.09 | 0.31 | 0.18 | 0.08 | 0.30 | 0.08 | 0.03 | 0.14 | 0.28 | 0.12 | 0.47 |
| S_97.5_9 | 4.77 | 4.77 | 4.77 | 25.34 | 7.90 | 0.12 | 0.12 | 0.12 | 0.14 | 0.07 | 0.24 | 0.14 | 0.07 | 0.24 | 0.14 | 0.07 | 0.24 |
| S_97.5_10 | -4.27 | -4.27 | -4.27 | 34.25 | -1.31 | 0.09 | 0.09 | 0.09 | 0.09 | 0.05 | 0.14 | 0.09 | 0.05 | 0.14 | 0.09 | 0.05 | 0.14 |
| S_97.5_12 | 7.60 | 7.60 | 7.60 | 63.78 | 5.05 | 0.04 | 0.04 | 0.04 | 0.05 | 0.00 | 0.11 | 0.05 | 0.00 | 0.11 | 0.05 | 0.00 | 0.11 |
| S_97.5_21 | -4.27 | -4.27 | -4.27 | 18.27 | -3.38 | 0.05 | 0.05 | 0.05 | 0.05 | 0.02 | 0.07 | 0.05 | 0.02 | 0.07 | 0.05 | 0.02 | 0.07 |
| S_97.5_22 | -4.27 | -4.27 | -4.27 | 14.43 | -3.69 | 0.04 | 0.04 | 0.04 | 0.04 | 0.02 | 0.07 | 0.04 | 0.02 | 0.07 | 0.04 | 0.02 | 0.07 |
