# Supplementary material for: Contrasting Patterns of Climatic Niche Divergence in Trebouxia—A Clade of Lichen-Forming Algae
Source: Front Microbiol. 2022 Feb 15;13:791546. doi: 10.3389/fmicb.2022.791546 (PMC8886231; doi:10.3389/fmicb.2022.791546)
Supplement: Supplementary file 6 [file Table_3.docx]

**Table S3** Summary data for absolute rates of climatic niche evolution for BIO5. OTU numbers are listed together with the modern mean value (ModMeanVal), the modern minimum value (ModMinVal), the modern maximum value (ModMaxVal), stem age from the MCC tree (Age), ancestral value based on the MCC tree (AncVal), the absolute rate of change using values from the MCC tree and the modern mean value (MCCAbsRateMean), the absolute rate of change using values from the MCC tree and the modern minimum value (MCCAbsRateMin), and the absolute rate of change using values from the MCC tree and the modern maximum value (MCCAbsRateMax). Rate estimates were also calculated across a distribution of 1000 trees derived from the posterior, and mean values presented, together with upper and lower limits of the 95% HPD. These include the: mean (MeanAbsRate_Mean), lower 95% HPD (MeanLower95Rate) and upper 95% HPD (MeanUpper95Rate) estimated rate of change using the modern mean value; mean (MinAbsRate_Mean), lower 95% HPD (MinLower95Rate) and upper 95% HPD (MinUpper95Rate) estimated rate of change using the modern minimum value; mean (MaxAbsRate_Mean), lower 95% HPD (MaxLower95Rate) and upper 95% HPD (MaxUpper95Rate) estimated rate of change using the modern maximum value.

|  | ModMeanVal | ModMinVal | ModMaxVal | Age | AncVal | MCCAbsRateMean | MCCAbsRateMin | MCCAbsRateMax | MeanAbsRate_Mean | MeanLower95Rate | MeanUpper95Rate | MinAbsRate_Mean | MinLower95Rate | MinUpper95Rate | MaxAbsRate_Mean | MaxLower95Rate | MaxUpper95Rate |
| --- | --- | --- | --- | --- | --- | --- | --- | --- | --- | --- | --- | --- | --- | --- | --- | --- | --- |
| A_97.5_1 | 11.19 | -14.93 | 40.97 | 37.54 | 18.01 | 0.18 | 0.88 | 0.61 | 0.21 | 0.10 | 0.36 | 0.96 | 0.42 | 1.65 | 0.64 | 0.14 | 1.37 |
| A_97.5_2 | 35.45 | 24.70 | 43.73 | 12.70 | 34.18 | 0.10 | 0.75 | 0.75 | 0.12 | 0.05 | 0.22 | 0.92 | 0.22 | 1.95 | 0.92 | 0.27 | 1.90 |
| A_97.5_3 | 32.50 | 18.23 | 42.57 | 21.25 | 32.92 | 0.02 | 0.69 | 0.45 | 0.04 | 0.00 | 0.11 | 0.74 | 0.27 | 1.30 | 0.48 | 0.25 | 0.79 |
| A_97.5_4 | 31.99 | 10.50 | 39.90 | 14.70 | 29.15 | 0.19 | 1.27 | 0.73 | 0.22 | 0.08 | 0.39 | 1.35 | 0.44 | 2.90 | 0.80 | 0.29 | 1.55 |
| A_97.5_5 | 30.91 | 10.50 | 37.50 | 15.49 | 35.98 | 0.33 | 1.64 | 0.10 | 0.34 | 0.12 | 0.67 | 1.65 | 0.64 | 3.13 | 0.09 | 0.02 | 0.17 |
| A_97.5_6 | 28.47 | -0.97 | 43.97 | 42.97 | 30.99 | 0.06 | 0.74 | 0.30 | 0.08 | 0.00 | 0.17 | 0.81 | 0.42 | 1.36 | 0.31 | 0.17 | 0.48 |
| A_97.5_7 | 32.86 | 22.63 | 41.03 | 16.54 | 33.26 | 0.02 | 0.64 | 0.47 | 0.03 | 0.00 | 0.06 | 0.82 | 0.29 | 1.50 | 0.60 | 0.23 | 1.09 |
| A_97.5_8 | 31.87 | 30.70 | 33.97 | 23.78 | 27.75 | 0.17 | 0.12 | 0.26 | 0.19 | 0.07 | 0.35 | 0.13 | 0.04 | 0.27 | 0.28 | 0.13 | 0.49 |
| A_97.5_9 | 29.37 | 23.13 | 42.63 | 23.78 | 27.75 | 0.07 | 0.19 | 0.63 | 0.08 | 0.00 | 0.19 | 0.21 | 0.05 | 0.42 | 0.67 | 0.32 | 1.12 |
| A_97.5_10 | 34.59 | 26.47 | 44.23 | 6.70 | 33.79 | 0.12 | 1.09 | 1.56 | 0.13 | 0.05 | 0.23 | 1.19 | 0.40 | 2.31 | 1.69 | 0.62 | 3.26 |
| A_97.5_11 | 34.79 | 21.60 | 43.73 | 22.68 | 33.92 | 0.04 | 0.54 | 0.43 | 0.05 | 0.01 | 0.10 | 0.55 | 0.20 | 1.08 | 0.46 | 0.22 | 0.80 |
| A_97.5_12 | 42.66 | 41.33 | 45.30 | 14.40 | 38.36 | 0.30 | 0.21 | 0.48 | 0.34 | 0.15 | 0.57 | 0.23 | 0.08 | 0.39 | 0.55 | 0.24 | 0.93 |
| A_97.5_13 | 33.35 | 27.17 | 44.20 | 6.70 | 33.79 | 0.07 | 0.99 | 1.55 | 0.07 | 0.01 | 0.16 | 1.07 | 0.36 | 2.09 | 1.68 | 0.59 | 3.22 |
| A_97.5_14 | 33.47 | 27.97 | 42.57 | 36.44 | 29.52 | 0.11 | 0.04 | 0.36 | 0.11 | 0.04 | 0.20 | 0.08 | 0.00 | 0.17 | 0.41 | 0.21 | 0.68 |
| A_97.5_15 | 35.43 | 27.57 | 42.77 | 22.68 | 33.92 | 0.07 | 0.28 | 0.39 | 0.08 | 0.04 | 0.12 | 0.29 | 0.08 | 0.58 | 0.42 | 0.20 | 0.73 |
| A_97.5_16 | 41.33 | 41.33 | 41.33 | 15.49 | 35.98 | 0.35 | 0.35 | 0.35 | 0.33 | 0.12 | 0.61 | 0.33 | 0.12 | 0.61 | 0.33 | 0.12 | 0.61 |
| A_97.5_17 | 35.03 | 35.03 | 35.03 | 79.97 | 29.06 | 0.07 | 0.07 | 0.07 | 0.08 | 0.04 | 0.14 | 0.08 | 0.04 | 0.14 | 0.08 | 0.04 | 0.14 |
| A_97.5_18 | 37.23 | 36.57 | 37.90 | 22.96 | 36.03 | 0.05 | 0.02 | 0.08 | 0.06 | 0.00 | 0.18 | 0.06 | 0.00 | 0.26 | 0.08 | 0.00 | 0.14 |
| A_97.5_19 | 41.33 | 41.33 | 41.33 | 26.18 | 35.55 | 0.22 | 0.22 | 0.22 | 0.22 | 0.11 | 0.34 | 0.22 | 0.11 | 0.34 | 0.22 | 0.11 | 0.34 |
| A_97.5_21 | 10.50 | 10.50 | 10.50 | 37.54 | 18.01 | 0.20 | 0.20 | 0.20 | 0.23 | 0.11 | 0.37 | 0.23 | 0.11 | 0.37 | 0.23 | 0.11 | 0.37 |
| A_97.5_22 | 29.33 | 29.33 | 29.33 | 18.00 | 36.55 | 0.40 | 0.40 | 0.40 | 0.43 | 0.17 | 0.73 | 0.43 | 0.17 | 0.73 | 0.43 | 0.17 | 0.73 |
| A_97.5_25 | 10.50 | 10.50 | 10.50 | 36.11 | 26.25 | 0.44 | 0.44 | 0.44 | 0.48 | 0.25 | 0.74 | 0.48 | 0.25 | 0.74 | 0.48 | 0.25 | 0.74 |
| A_97.5_26 | 38.40 | 34.03 | 42.77 | 29.31 | 32.54 | 0.20 | 0.05 | 0.35 | 0.21 | 0.12 | 0.33 | 0.06 | 0.02 | 0.10 | 0.37 | 0.20 | 0.58 |
| A_97.5_28 | 33.57 | 33.53 | 33.63 | 9.90 | 33.62 | 0.01 | 0.01 | 0.00 | 0.01 | 0.00 | 0.03 | 0.01 | 0.00 | 0.03 | 0.01 | 0.00 | 0.02 |
| A_97.5_29 | 34.98 | 33.80 | 38.53 | 25.95 | 30.75 | 0.16 | 0.12 | 0.30 | 0.13 | 0.04 | 0.24 | 0.08 | 0.00 | 0.17 | 0.29 | 0.13 | 0.46 |
| A_97.5_30 | 33.58 | 31.10 | 37.83 | 12.92 | 33.47 | 0.01 | 0.18 | 0.34 | 0.02 | 0.00 | 0.04 | 0.20 | 0.08 | 0.35 | 0.40 | 0.18 | 0.70 |
| A_97.5_31 | 28.73 | 28.73 | 28.73 | 29.31 | 32.54 | 0.13 | 0.13 | 0.13 | 0.13 | 0.04 | 0.25 | 0.13 | 0.04 | 0.25 | 0.13 | 0.04 | 0.25 |
| A_97.5_32 | 33.13 | 24.93 | 41.33 | 18.46 | 36.16 | 0.16 | 0.61 | 0.28 | 0.15 | 0.00 | 0.30 | 0.58 | 0.17 | 1.05 | 0.28 | 0.13 | 0.47 |
| A_97.5_33 | 41.33 | 41.33 | 41.33 | 14.40 | 38.36 | 0.21 | 0.21 | 0.21 | 0.24 | 0.08 | 0.42 | 0.24 | 0.08 | 0.42 | 0.24 | 0.08 | 0.42 |
| A_97.5_35 | 26.47 | 26.47 | 26.47 | 23.67 | 27.67 | 0.05 | 0.05 | 0.05 | 0.05 | 0.00 | 0.12 | 0.05 | 0.00 | 0.12 | 0.05 | 0.00 | 0.12 |
| A_97.5_37 | 40.07 | 40.07 | 40.07 | 9.21 | 33.56 | 0.71 | 0.71 | 0.71 | 0.86 | 0.21 | 1.76 | 0.86 | 0.21 | 1.76 | 0.86 | 0.21 | 1.76 |
| A_97.5_38 | 4.10 | 4.10 | 4.10 | 21.81 | 25.30 | 0.97 | 0.97 | 0.97 | 1.05 | 0.44 | 1.75 | 1.05 | 0.44 | 1.75 | 1.05 | 0.44 | 1.75 |
| A_97.5_39 | 25.97 | 25.97 | 25.97 | 9.21 | 33.56 | 0.82 | 0.82 | 0.82 | 0.95 | 0.25 | 1.94 | 0.95 | 0.25 | 1.94 | 0.95 | 0.25 | 1.94 |
| A_97.5_40 | 41.03 | 41.03 | 41.03 | 21.81 | 25.30 | 0.72 | 0.72 | 0.72 | 0.80 | 0.30 | 1.51 | 0.80 | 0.30 | 1.51 | 0.80 | 0.30 | 1.51 |
| A_97.5_43 | 38.80 | 38.80 | 38.80 | 21.25 | 32.92 | 0.28 | 0.28 | 0.28 | 0.29 | 0.15 | 0.47 | 0.29 | 0.15 | 0.47 | 0.29 | 0.15 | 0.47 |
| A_97.5_46 | 33.63 | 33.63 | 33.63 | 17.78 | 33.40 | 0.01 | 0.01 | 0.01 | 0.02 | 0.00 | 0.04 | 0.02 | 0.00 | 0.04 | 0.02 | 0.00 | 0.04 |
| A_97.5_47 | 33.27 | 33.27 | 33.27 | 50.58 | 29.40 | 0.08 | 0.08 | 0.08 | 0.09 | 0.04 | 0.14 | 0.09 | 0.04 | 0.14 | 0.09 | 0.04 | 0.14 |
| A_97.5_48 | 33.63 | 33.63 | 33.63 | 12.70 | 34.18 | 0.04 | 0.04 | 0.04 | 0.06 | 0.00 | 0.16 | 0.06 | 0.00 | 0.16 | 0.06 | 0.00 | 0.16 |
| A_97.5_49 | 28.73 | 28.73 | 28.73 | 14.70 | 29.15 | 0.03 | 0.03 | 0.03 | 0.05 | 0.00 | 0.14 | 0.05 | 0.00 | 0.14 | 0.05 | 0.00 | 0.14 |
| A_97.5_62 | 40.53 | 40.53 | 40.53 | 18.46 | 36.16 | 0.24 | 0.24 | 0.24 | 0.23 | 0.11 | 0.39 | 0.23 | 0.11 | 0.39 | 0.23 | 0.11 | 0.39 |
| A_97.5_64 | 26.43 | 26.43 | 26.43 | 32.14 | 30.13 | 0.11 | 0.11 | 0.11 | 0.10 | 0.00 | 0.20 | 0.10 | 0.00 | 0.20 | 0.10 | 0.00 | 0.20 |
| A_97.5_65 | 33.70 | 33.70 | 33.70 | 25.95 | 30.75 | 0.11 | 0.11 | 0.11 | 0.08 | 0.00 | 0.16 | 0.08 | 0.00 | 0.16 | 0.08 | 0.00 | 0.16 |
| A_97.5_68 | 33.83 | 33.83 | 33.83 | 15.06 | 33.52 | 0.02 | 0.02 | 0.02 | 0.03 | 0.01 | 0.05 | 0.03 | 0.01 | 0.05 | 0.03 | 0.01 | 0.05 |
| A_97.5_70 | 33.83 | 33.83 | 33.83 | 15.06 | 33.52 | 0.02 | 0.02 | 0.02 | 0.03 | 0.01 | 0.05 | 0.03 | 0.01 | 0.05 | 0.03 | 0.01 | 0.05 |
| A_97.5_72 | -0.97 | -0.97 | -0.97 | 41.15 | 23.57 | 0.60 | 0.60 | 0.60 | 0.63 | 0.33 | 0.99 | 0.63 | 0.33 | 0.99 | 0.63 | 0.33 | 0.99 |
| I_97.5_1 | 29.73 | 5.07 | 41.33 | 11.39 | 22.23 | 0.66 | 1.51 | 1.68 | 0.77 | 0.17 | 1.70 | 1.72 | 0.61 | 3.15 | 1.94 | 0.55 | 3.93 |
| I_97.5_2 | 24.00 | 24.00 | 24.00 | 52.57 | 29.75 | 0.11 | 0.11 | 0.11 | 0.12 | 0.06 | 0.19 | 0.12 | 0.06 | 0.19 | 0.12 | 0.06 | 0.19 |
| I_97.5_3 | 40.40 | 39.47 | 41.33 | 36.38 | 33.41 | 0.19 | 0.17 | 0.22 | 0.19 | 0.09 | 0.31 | 0.17 | 0.08 | 0.27 | 0.22 | 0.11 | 0.35 |
| I_97.5_4 | 32.14 | 24.70 | 40.63 | 24.85 | 27.21 | 0.20 | 0.10 | 0.54 | 0.22 | 0.06 | 0.43 | 0.10 | 0.04 | 0.18 | 0.59 | 0.25 | 1.06 |
| I_97.5_6 | 26.71 | 18.87 | 41.33 | 40.05 | 31.90 | 0.13 | 0.33 | 0.24 | 0.08 | 0.01 | 0.18 | 0.29 | 0.14 | 0.46 | 0.31 | 0.14 | 0.52 |
| I_97.5_8 | 41.33 | 41.33 | 41.33 | 36.38 | 33.41 | 0.22 | 0.22 | 0.22 | 0.21 | 0.11 | 0.35 | 0.21 | 0.11 | 0.35 | 0.21 | 0.11 | 0.35 |
| I_97.5_11 | 18.23 | 18.23 | 18.23 | 15.25 | 27.61 | 0.61 | 0.61 | 0.61 | 0.63 | 0.22 | 1.17 | 0.63 | 0.22 | 1.17 | 0.63 | 0.22 | 1.17 |
| I_97.5_12 | 10.50 | 10.50 | 10.50 | 11.39 | 22.23 | 1.03 | 1.03 | 1.03 | 1.17 | 0.51 | 2.15 | 1.17 | 0.51 | 2.15 | 1.17 | 0.51 | 2.15 |
| I_97.5_13 | 33.63 | 33.63 | 33.63 | 32.97 | 29.73 | 0.12 | 0.12 | 0.12 | 0.12 | 0.05 | 0.22 | 0.12 | 0.05 | 0.22 | 0.12 | 0.05 | 0.22 |
| I_97.5_16 | 35.17 | 35.17 | 35.17 | 15.25 | 27.61 | 0.50 | 0.50 | 0.50 | 0.50 | 0.13 | 1.01 | 0.50 | 0.13 | 1.01 | 0.50 | 0.13 | 1.01 |
| C_97.5_1 | 35.84 | 32.80 | 37.37 | 16.69 | 39.41 | 0.21 | 0.40 | 0.12 | 0.25 | 0.06 | 0.53 | 0.46 | 0.13 | 0.98 | 0.14 | 0.03 | 0.31 |
| C_97.5_3 | 34.45 | 20.53 | 39.33 | 9.98 | 36.21 | 0.18 | 1.57 | 0.31 | 0.21 | 0.06 | 0.42 | 1.86 | 0.48 | 3.91 | 0.38 | 0.09 | 0.80 |
| C_97.5_5 | 38.04 | 37.37 | 39.10 | 10.16 | 37.72 | 0.03 | 0.03 | 0.14 | 0.03 | 0.00 | 0.08 | 0.04 | 0.01 | 0.09 | 0.15 | 0.04 | 0.33 |
| C_97.5_7 | 37.37 | 37.37 | 37.37 | 10.16 | 37.72 | 0.03 | 0.03 | 0.03 | 0.04 | 0.01 | 0.09 | 0.04 | 0.01 | 0.09 | 0.04 | 0.01 | 0.09 |
| C_97.5_8 | 42.17 | 37.33 | 45.23 | 42.94 | 39.82 | 0.05 | 0.06 | 0.13 | 0.06 | 0.03 | 0.09 | 0.07 | 0.01 | 0.13 | 0.13 | 0.07 | 0.20 |
| C_97.5_9 | 37.37 | 36.70 | 38.43 | 23.38 | 36.90 | 0.02 | 0.01 | 0.07 | 0.03 | 0.00 | 0.06 | 0.01 | 0.00 | 0.02 | 0.08 | 0.02 | 0.15 |
| C_97.5_10 | 43.27 | 43.20 | 43.33 | 16.69 | 39.41 | 0.23 | 0.23 | 0.23 | 0.27 | 0.09 | 0.55 | 0.26 | 0.09 | 0.54 | 0.27 | 0.09 | 0.56 |
| C_97.5_13 | 39.10 | 39.10 | 39.10 | 40.15 | 37.80 | 0.03 | 0.03 | 0.03 | 0.03 | 0.01 | 0.05 | 0.03 | 0.01 | 0.05 | 0.03 | 0.01 | 0.05 |
| C_97.5_14 | 34.23 | 34.23 | 34.23 | 84.80 | 34.33 | 0.00 | 0.00 | 0.00 | 0.01 | 0.00 | 0.03 | 0.01 | 0.00 | 0.03 | 0.01 | 0.00 | 0.03 |
| C_97.5_15 | 42.82 | 41.53 | 44.10 | 42.94 | 39.82 | 0.07 | 0.04 | 0.10 | 0.07 | 0.04 | 0.11 | 0.04 | 0.02 | 0.07 | 0.10 | 0.06 | 0.16 |
| C_97.5_17 | 37.23 | 37.23 | 37.23 | 36.31 | 37.35 | 0.00 | 0.00 | 0.00 | 0.01 | 0.00 | 0.02 | 0.01 | 0.00 | 0.02 | 0.01 | 0.00 | 0.02 |
| C_97.5_18 | 37.47 | 37.47 | 37.47 | 9.98 | 36.21 | 0.13 | 0.13 | 0.13 | 0.15 | 0.03 | 0.34 | 0.15 | 0.03 | 0.34 | 0.15 | 0.03 | 0.34 |
| C_97.5_20 | 37.47 | 37.47 | 37.47 | 9.00 | 37.45 | 0.00 | 0.00 | 0.00 | 0.00 | 0.00 | 0.01 | 0.00 | 0.00 | 0.01 | 0.00 | 0.00 | 0.01 |
| C_97.5_21 | 37.47 | 37.47 | 37.47 | 9.00 | 37.45 | 0.00 | 0.00 | 0.00 | 0.00 | 0.00 | 0.01 | 0.00 | 0.00 | 0.01 | 0.00 | 0.00 | 0.01 |
| S_97.5_1 | 25.12 | -0.67 | 44.07 | 15.72 | 28.49 | 0.21 | 1.85 | 0.99 | 0.19 | 0.09 | 0.33 | 1.95 | 1.02 | 3.22 | 1.11 | 0.52 | 1.83 |
| S_97.5_2 | 34.72 | 22.83 | 44.57 | 86.68 | 28.03 | 0.08 | 0.06 | 0.19 | 0.09 | 0.03 | 0.15 | 0.07 | 0.03 | 0.13 | 0.22 | 0.11 | 0.35 |
| S_97.5_3 | 34.23 | 25.57 | 39.00 | 28.08 | 30.55 | 0.13 | 0.18 | 0.30 | 0.12 | 0.06 | 0.20 | 0.23 | 0.08 | 0.48 | 0.31 | 0.16 | 0.51 |
| S_97.5_4 | 29.48 | 22.13 | 36.30 | 49.85 | 28.89 | 0.01 | 0.14 | 0.15 | 0.01 | 0.00 | 0.04 | 0.15 | 0.06 | 0.24 | 0.15 | 0.08 | 0.23 |
| S_97.5_6 | 24.38 | 24.37 | 24.40 | 19.53 | 28.74 | 0.22 | 0.22 | 0.22 | 0.22 | 0.11 | 0.36 | 0.22 | 0.11 | 0.36 | 0.22 | 0.11 | 0.36 |
| S_97.5_7 | 10.50 | 10.50 | 10.50 | 14.43 | 11.23 | 0.05 | 0.05 | 0.05 | 0.06 | 0.03 | 0.09 | 0.06 | 0.03 | 0.09 | 0.06 | 0.03 | 0.09 |
| S_97.5_8 | 30.87 | 25.83 | 35.90 | 15.72 | 28.49 | 0.15 | 0.17 | 0.47 | 0.12 | 0.03 | 0.24 | 0.18 | 0.09 | 0.29 | 0.41 | 0.17 | 0.70 |
| S_97.5_9 | 36.30 | 36.30 | 36.30 | 25.34 | 30.41 | 0.23 | 0.23 | 0.23 | 0.24 | 0.12 | 0.39 | 0.24 | 0.12 | 0.39 | 0.24 | 0.12 | 0.39 |
| S_97.5_10 | 10.50 | 10.50 | 10.50 | 34.25 | 14.24 | 0.11 | 0.11 | 0.11 | 0.11 | 0.05 | 0.18 | 0.11 | 0.05 | 0.18 | 0.11 | 0.05 | 0.18 |
| S_97.5_12 | 21.60 | 21.60 | 21.60 | 63.78 | 22.29 | 0.01 | 0.01 | 0.01 | 0.02 | 0.00 | 0.06 | 0.02 | 0.00 | 0.06 | 0.02 | 0.00 | 0.06 |
| S_97.5_21 | 10.50 | 10.50 | 10.50 | 18.27 | 11.63 | 0.06 | 0.06 | 0.06 | 0.06 | 0.03 | 0.10 | 0.06 | 0.03 | 0.10 | 0.06 | 0.03 | 0.10 |
| S_97.5_22 | 10.50 | 10.50 | 10.50 | 14.43 | 11.23 | 0.05 | 0.05 | 0.05 | 0.05 | 0.03 | 0.09 | 0.05 | 0.03 | 0.09 | 0.05 | 0.03 | 0.09 |
