# Supplementary material for: Contrasting Patterns of Climatic Niche Divergence in Trebouxia—A Clade of Lichen-Forming Algae
Source: Front Microbiol. 2022 Feb 15;13:791546. doi: 10.3389/fmicb.2022.791546 (PMC8886231; doi:10.3389/fmicb.2022.791546)
Supplement: Supplementary file 7 [file Table_4.docx]

**Table S4** Summary data for absolute rates of climatic niche evolution for BIO6. OTU numbers are listed together with the modern mean value (ModMeanVal), the modern minimum value (ModMinVal), the modern maximum value (ModMaxVal), stem age from the MCC tree (Age), ancestral value based on the MCC tree (AncVal), the absolute rate of change using values from the MCC tree and the modern mean value (MCCAbsRateMean), the absolute rate of change using values from the MCC tree and the modern minimum value (MCCAbsRateMin), and the absolute rate of change using values from the MCC tree and the modern maximum value (MCCAbsRateMax). Rate estimates were also calculated across a distribution of 1000 trees derived from the posterior, and mean values presented, together with upper and lower limits of the 95% HPD. These include the: mean (MeanAbsRate_Mean), lower 95% HPD (MeanLower95Rate) and upper 95% HPD (MeanUpper95Rate) estimated rate of change using the modern mean value; mean (MinAbsRate_Mean), lower 95% HPD (MinLower95Rate) and upper 95% HPD (MinUpper95Rate) estimated rate of change using the modern minimum value; mean (MaxAbsRate_Mean), lower 95% HPD (MaxLower95Rate) and upper 95% HPD (MaxUpper95Rate) estimated rate of change using the modern maximum value.

|  | ModMeanVal | ModMinVal | ModMaxVal | Age | AncVal | MCCAbsRateMean | MCCAbsRateMin | MCCAbsRateMax | MeanAbsRate_Mean | MeanLower95Rate | MeanUpper95Rate | MinAbsRate_Mean | MinLower95Rate | MinUpper95Rate | MaxAbsRate_Mean | MaxLower95Rate | MaxUpper95Rate |
| --- | --- | --- | --- | --- | --- | --- | --- | --- | --- | --- | --- | --- | --- | --- | --- | --- | --- |
| A_97.5_1 | -31.78 | -52.83 | -3.73 | 37.54 | -21.57 | 0.27 | 0.83 | 0.48 | 0.30 | 0.16 | 0.47 | 0.91 | 0.39 | 1.57 | 0.50 | 0.10 | 1.09 |
| A_97.5_2 | -23.88 | -38.83 | -2.43 | 12.70 | -22.12 | 0.14 | 1.32 | 1.55 | 0.16 | 0.08 | 0.26 | 1.61 | 0.51 | 3.28 | 1.91 | 0.50 | 4.10 |
| A_97.5_3 | -3.89 | -41.90 | 16.57 | 21.25 | -3.06 | 0.04 | 1.83 | 0.92 | 0.09 | 0.00 | 0.24 | 1.95 | 0.81 | 3.47 | 0.97 | 0.50 | 1.64 |
| A_97.5_4 | -19.94 | -34.40 | 11.10 | 14.70 | -23.74 | 0.26 | 0.72 | 2.37 | 0.29 | 0.00 | 0.82 | 0.77 | 0.31 | 1.44 | 2.55 | 0.75 | 5.48 |
| A_97.5_5 | -13.22 | -23.77 | -6.63 | 15.49 | -4.26 | 0.58 | 1.26 | 0.15 | 0.56 | 0.19 | 1.06 | 1.24 | 0.43 | 2.30 | 0.14 | 0.00 | 0.27 |
| A_97.5_6 | -0.67 | -46.50 | 16.93 | 42.97 | -6.98 | 0.15 | 0.92 | 0.56 | 0.15 | 0.06 | 0.26 | 1.00 | 0.49 | 1.66 | 0.59 | 0.32 | 0.90 |
| A_97.5_7 | -22.88 | -34.20 | 7.63 | 16.54 | -21.45 | 0.09 | 0.77 | 1.76 | 0.07 | 0.00 | 0.12 | 0.93 | 0.38 | 1.62 | 2.28 | 0.75 | 4.23 |
| A_97.5_8 | 1.79 | 1.07 | 3.23 | 23.78 | -9.55 | 0.48 | 0.45 | 0.54 | 0.52 | 0.24 | 0.85 | 0.49 | 0.24 | 0.83 | 0.58 | 0.27 | 0.96 |
| A_97.5_9 | -11.70 | -45.40 | 7.00 | 23.78 | -9.55 | 0.09 | 1.51 | 0.70 | 0.10 | 0.00 | 0.26 | 1.61 | 0.70 | 2.73 | 0.75 | 0.36 | 1.23 |
| A_97.5_10 | -15.55 | -32.90 | 8.53 | 6.70 | -19.08 | 0.53 | 2.06 | 4.12 | 0.57 | 0.23 | 1.00 | 2.24 | 0.76 | 4.47 | 4.47 | 1.67 | 8.60 |
| A_97.5_11 | -16.38 | -34.20 | -3.73 | 22.68 | -18.87 | 0.11 | 0.68 | 0.67 | 0.11 | 0.02 | 0.22 | 0.70 | 0.31 | 1.31 | 0.68 | 0.26 | 1.26 |
| A_97.5_12 | 5.47 | 4.43 | 7.53 | 14.40 | -1.48 | 0.48 | 0.41 | 0.63 | 0.56 | 0.21 | 1.01 | 0.48 | 0.17 | 0.87 | 0.72 | 0.28 | 1.29 |
| A_97.5_13 | -19.27 | -31.97 | 8.53 | 6.70 | -19.08 | 0.03 | 1.92 | 4.12 | 0.08 | 0.00 | 0.22 | 2.09 | 0.64 | 4.10 | 4.46 | 1.67 | 8.60 |
| A_97.5_14 | -3.31 | -19.80 | 16.27 | 36.44 | -10.10 | 0.19 | 0.27 | 0.72 | 0.20 | 0.02 | 0.38 | 0.35 | 0.12 | 0.65 | 0.85 | 0.41 | 1.42 |
| A_97.5_15 | -20.18 | -31.77 | -2.43 | 22.68 | -18.87 | 0.06 | 0.57 | 0.72 | 0.06 | 0.00 | 0.13 | 0.60 | 0.26 | 1.11 | 0.78 | 0.30 | 1.41 |
| A_97.5_16 | 4.43 | 4.43 | 4.43 | 15.49 | -4.26 | 0.56 | 0.56 | 0.56 | 0.58 | 0.23 | 1.09 | 0.58 | 0.23 | 1.09 | 0.58 | 0.23 | 1.09 |
| A_97.5_17 | -6.53 | -6.53 | -6.53 | 79.97 | -12.39 | 0.07 | 0.07 | 0.07 | 0.08 | 0.03 | 0.15 | 0.08 | 0.03 | 0.15 | 0.08 | 0.03 | 0.15 |
| A_97.5_18 | -8.53 | -8.77 | -8.30 | 22.96 | -4.97 | 0.16 | 0.17 | 0.14 | 0.28 | 0.07 | 0.72 | 0.29 | 0.08 | 0.75 | 0.27 | 0.05 | 0.69 |
| A_97.5_19 | 4.43 | 4.43 | 4.43 | 26.18 | -0.37 | 0.18 | 0.18 | 0.18 | 0.28 | 0.11 | 0.49 | 0.28 | 0.11 | 0.49 | 0.28 | 0.11 | 0.49 |
| A_97.5_21 | -23.77 | -23.77 | -23.77 | 37.54 | -21.57 | 0.06 | 0.06 | 0.06 | 0.09 | 0.00 | 0.19 | 0.09 | 0.00 | 0.19 | 0.09 | 0.00 | 0.19 |
| A_97.5_22 | -19.77 | -19.77 | -19.77 | 18.00 | -4.70 | 0.84 | 0.84 | 0.84 | 0.92 | 0.45 | 1.56 | 0.92 | 0.45 | 1.56 | 0.92 | 0.45 | 1.56 |
| A_97.5_25 | -23.77 | -23.77 | -23.77 | 36.11 | -19.41 | 0.12 | 0.12 | 0.12 | 0.13 | 0.06 | 0.20 | 0.13 | 0.06 | 0.20 | 0.13 | 0.06 | 0.20 |
| A_97.5_26 | -13.15 | -23.87 | -2.43 | 29.31 | -13.92 | 0.03 | 0.34 | 0.39 | 0.06 | 0.00 | 0.14 | 0.35 | 0.13 | 0.65 | 0.42 | 0.24 | 0.65 |
| A_97.5_28 | -22.81 | -22.93 | -22.57 | 9.90 | -20.68 | 0.22 | 0.23 | 0.19 | 0.26 | 0.08 | 0.50 | 0.27 | 0.09 | 0.53 | 0.23 | 0.07 | 0.46 |
| A_97.5_29 | -20.99 | -22.13 | -17.57 | 25.95 | -17.71 | 0.13 | 0.17 | 0.01 | 0.17 | 0.07 | 0.31 | 0.21 | 0.10 | 0.40 | 0.02 | 0.00 | 0.08 |
| A_97.5_30 | -25.47 | -32.90 | -16.40 | 12.92 | -21.53 | 0.31 | 0.88 | 0.40 | 0.30 | 0.14 | 0.50 | 0.96 | 0.46 | 1.67 | 0.51 | 0.17 | 0.93 |
| A_97.5_31 | -1.40 | -1.40 | -1.40 | 29.31 | -13.92 | 0.43 | 0.43 | 0.43 | 0.45 | 0.25 | 0.68 | 0.45 | 0.25 | 0.68 | 0.45 | 0.25 | 0.68 |
| A_97.5_32 | -9.97 | -24.37 | 4.43 | 18.46 | -5.36 | 0.25 | 1.03 | 0.53 | 0.26 | 0.10 | 0.48 | 1.02 | 0.38 | 1.74 | 0.49 | 0.18 | 0.88 |
| A_97.5_33 | 4.43 | 4.43 | 4.43 | 14.40 | -1.48 | 0.41 | 0.41 | 0.41 | 0.45 | 0.17 | 0.79 | 0.45 | 0.17 | 0.79 | 0.45 | 0.17 | 0.79 |
| A_97.5_35 | -9.50 | -9.50 | -9.50 | 23.67 | -19.68 | 0.43 | 0.43 | 0.43 | 0.44 | 0.17 | 0.81 | 0.44 | 0.17 | 0.81 | 0.44 | 0.17 | 0.81 |
| A_97.5_37 | 7.60 | 7.60 | 7.60 | 9.21 | 8.47 | 0.09 | 0.09 | 0.09 | 0.17 | 0.00 | 0.53 | 0.17 | 0.00 | 0.53 | 0.17 | 0.00 | 0.53 |
| A_97.5_38 | -23.47 | -23.47 | -23.47 | 21.81 | -18.61 | 0.22 | 0.22 | 0.22 | 0.24 | 0.11 | 0.43 | 0.24 | 0.11 | 0.43 | 0.24 | 0.11 | 0.43 |
| A_97.5_39 | 14.13 | 14.13 | 14.13 | 9.21 | 8.47 | 0.61 | 0.61 | 0.61 | 0.71 | 0.33 | 1.26 | 0.71 | 0.33 | 1.26 | 0.71 | 0.33 | 1.26 |
| A_97.5_40 | -14.40 | -14.40 | -14.40 | 21.81 | -18.61 | 0.19 | 0.19 | 0.19 | 0.21 | 0.08 | 0.39 | 0.21 | 0.08 | 0.39 | 0.21 | 0.08 | 0.39 |
| A_97.5_43 | 10.80 | 10.80 | 10.80 | 21.25 | -3.06 | 0.65 | 0.65 | 0.65 | 0.68 | 0.36 | 1.13 | 0.68 | 0.36 | 1.13 | 0.68 | 0.36 | 1.13 |
| A_97.5_46 | -22.57 | -22.57 | -22.57 | 17.78 | -21.97 | 0.03 | 0.03 | 0.03 | 0.04 | 0.00 | 0.09 | 0.04 | 0.00 | 0.09 | 0.04 | 0.00 | 0.09 |
| A_97.5_47 | -24.37 | -24.37 | -24.37 | 50.58 | -17.78 | 0.13 | 0.13 | 0.13 | 0.14 | 0.08 | 0.22 | 0.14 | 0.08 | 0.22 | 0.14 | 0.08 | 0.22 |
| A_97.5_48 | -22.57 | -22.57 | -22.57 | 12.70 | -22.12 | 0.03 | 0.03 | 0.03 | 0.05 | 0.00 | 0.11 | 0.05 | 0.00 | 0.11 | 0.05 | 0.00 | 0.11 |
| A_97.5_49 | -34.20 | -34.20 | -34.20 | 14.70 | -23.74 | 0.71 | 0.71 | 0.71 | 0.76 | 0.31 | 1.41 | 0.76 | 0.31 | 1.41 | 0.76 | 0.31 | 1.41 |
| A_97.5_62 | -6.00 | -6.00 | -6.00 | 18.46 | -5.36 | 0.03 | 0.03 | 0.03 | 0.07 | 0.00 | 0.19 | 0.07 | 0.00 | 0.19 | 0.07 | 0.00 | 0.19 |
| A_97.5_64 | -26.70 | -26.70 | -26.70 | 32.14 | -9.73 | 0.53 | 0.53 | 0.53 | 0.51 | 0.22 | 0.89 | 0.51 | 0.22 | 0.89 | 0.51 | 0.22 | 0.89 |
| A_97.5_65 | -12.73 | -12.73 | -12.73 | 25.95 | -17.71 | 0.19 | 0.19 | 0.19 | 0.19 | 0.09 | 0.34 | 0.19 | 0.09 | 0.34 | 0.19 | 0.09 | 0.34 |
| A_97.5_68 | -23.57 | -23.57 | -23.57 | 15.06 | -22.39 | 0.08 | 0.08 | 0.08 | 0.09 | 0.05 | 0.15 | 0.09 | 0.05 | 0.15 | 0.09 | 0.05 | 0.15 |
| A_97.5_70 | -23.57 | -23.57 | -23.57 | 15.06 | -22.39 | 0.08 | 0.08 | 0.08 | 0.09 | 0.05 | 0.15 | 0.09 | 0.05 | 0.15 | 0.09 | 0.05 | 0.15 |
| A_97.5_72 | -46.53 | -46.53 | -46.53 | 41.15 | -16.26 | 0.74 | 0.74 | 0.74 | 0.77 | 0.39 | 1.22 | 0.77 | 0.39 | 1.22 | 0.77 | 0.39 | 1.22 |
| I_97.5_1 | -20.86 | -40.57 | 16.57 | 11.39 | -20.40 | 0.04 | 1.77 | 3.25 | 0.08 | 0.00 | 0.20 | 2.04 | 0.76 | 3.77 | 3.72 | 1.06 | 7.36 |
| I_97.5_2 | 2.60 | 2.60 | 2.60 | 52.57 | -5.68 | 0.16 | 0.16 | 0.16 | 0.19 | 0.09 | 0.35 | 0.19 | 0.09 | 0.35 | 0.19 | 0.09 | 0.35 |
| I_97.5_3 | 0.18 | -4.07 | 4.43 | 36.38 | -3.45 | 0.10 | 0.02 | 0.22 | 0.11 | 0.03 | 0.22 | 0.04 | 0.00 | 0.11 | 0.23 | 0.12 | 0.39 |
| I_97.5_4 | -19.48 | -27.80 | -12.07 | 24.85 | -15.90 | 0.14 | 0.48 | 0.15 | 0.17 | 0.05 | 0.33 | 0.53 | 0.28 | 0.82 | 0.15 | 0.00 | 0.39 |
| I_97.5_6 | -1.72 | -5.30 | 4.43 | 40.05 | -4.61 | 0.07 | 0.02 | 0.23 | 0.11 | 0.02 | 0.21 | 0.03 | 0.00 | 0.10 | 0.27 | 0.12 | 0.43 |
| I_97.5_8 | 4.43 | 4.43 | 4.43 | 36.38 | -3.45 | 0.22 | 0.22 | 0.22 | 0.22 | 0.11 | 0.36 | 0.22 | 0.11 | 0.36 | 0.22 | 0.11 | 0.36 |
| I_97.5_11 | -2.20 | -2.20 | -2.20 | 15.25 | -5.21 | 0.20 | 0.20 | 0.20 | 0.21 | 0.09 | 0.37 | 0.21 | 0.09 | 0.37 | 0.21 | 0.09 | 0.37 |
| I_97.5_12 | -23.77 | -23.77 | -23.77 | 11.39 | -20.40 | 0.30 | 0.30 | 0.30 | 0.35 | 0.17 | 0.54 | 0.35 | 0.17 | 0.54 | 0.35 | 0.17 | 0.54 |
| I_97.5_13 | -13.77 | -13.77 | -13.77 | 32.97 | -7.87 | 0.18 | 0.18 | 0.18 | 0.20 | 0.10 | 0.33 | 0.20 | 0.10 | 0.33 | 0.20 | 0.10 | 0.33 |
| I_97.5_16 | -5.93 | -5.93 | -5.93 | 15.25 | -5.21 | 0.05 | 0.05 | 0.05 | 0.05 | 0.00 | 0.14 | 0.05 | 0.00 | 0.14 | 0.05 | 0.00 | 0.14 |
| C_97.5_1 | 5.63 | 0.83 | 13.40 | 16.69 | 9.15 | 0.21 | 0.50 | 0.25 | 0.25 | 0.04 | 0.59 | 0.58 | 0.14 | 1.30 | 0.29 | 0.12 | 0.55 |
| C_97.5_3 | -0.63 | -5.90 | 13.17 | 9.98 | 2.32 | 0.30 | 0.82 | 1.09 | 0.35 | 0.08 | 0.76 | 0.98 | 0.23 | 2.06 | 1.29 | 0.32 | 2.67 |
| C_97.5_5 | 5.33 | 0.83 | 8.53 | 10.16 | 4.46 | 0.09 | 0.36 | 0.40 | 0.10 | 0.02 | 0.23 | 0.41 | 0.11 | 0.87 | 0.46 | 0.12 | 0.99 |
| C_97.5_7 | 3.32 | 0.83 | 5.80 | 10.16 | 4.46 | 0.11 | 0.36 | 0.13 | 0.13 | 0.03 | 0.26 | 0.41 | 0.11 | 0.87 | 0.15 | 0.03 | 0.33 |
| C_97.5_8 | 5.32 | 1.43 | 11.30 | 42.94 | 5.47 | 0.00 | 0.09 | 0.14 | 0.01 | 0.00 | 0.03 | 0.10 | 0.04 | 0.19 | 0.15 | 0.08 | 0.23 |
| C_97.5_9 | -1.88 | -3.83 | 0.43 | 23.38 | 1.47 | 0.14 | 0.23 | 0.04 | 0.16 | 0.07 | 0.28 | 0.25 | 0.11 | 0.45 | 0.05 | 0.02 | 0.08 |
| C_97.5_10 | 14.62 | 14.27 | 14.97 | 16.69 | 9.15 | 0.33 | 0.31 | 0.35 | 0.38 | 0.15 | 0.73 | 0.35 | 0.14 | 0.68 | 0.40 | 0.16 | 0.78 |
| C_97.5_13 | 8.53 | 8.53 | 8.53 | 40.15 | 5.26 | 0.08 | 0.08 | 0.08 | 0.09 | 0.05 | 0.14 | 0.09 | 0.05 | 0.14 | 0.09 | 0.05 | 0.14 |
| C_97.5_14 | 8.33 | 8.33 | 8.33 | 84.80 | 0.20 | 0.10 | 0.10 | 0.10 | 0.09 | 0.05 | 0.15 | 0.09 | 0.05 | 0.15 | 0.09 | 0.05 | 0.15 |
| C_97.5_15 | 7.38 | 3.37 | 11.40 | 42.94 | 5.47 | 0.04 | 0.05 | 0.14 | 0.05 | 0.02 | 0.08 | 0.05 | 0.01 | 0.11 | 0.15 | 0.08 | 0.24 |
| C_97.5_17 | 5.63 | 5.63 | 5.63 | 36.31 | 5.14 | 0.01 | 0.01 | 0.01 | 0.02 | 0.00 | 0.04 | 0.02 | 0.00 | 0.04 | 0.02 | 0.00 | 0.04 |
| C_97.5_18 | 5.90 | 5.90 | 5.90 | 9.98 | 2.32 | 0.36 | 0.36 | 0.36 | 0.43 | 0.11 | 0.86 | 0.43 | 0.11 | 0.86 | 0.43 | 0.11 | 0.86 |
| C_97.5_20 | 5.90 | 5.90 | 5.90 | 9.00 | 5.79 | 0.01 | 0.01 | 0.01 | 0.02 | 0.00 | 0.04 | 0.02 | 0.00 | 0.04 | 0.02 | 0.00 | 0.04 |
| C_97.5_21 | 5.90 | 5.90 | 5.90 | 9.00 | 5.79 | 0.01 | 0.01 | 0.01 | 0.02 | 0.00 | 0.04 | 0.02 | 0.00 | 0.04 | 0.02 | 0.00 | 0.04 |
| S_97.5_1 | -15.90 | -46.23 | 14.20 | 15.72 | -11.31 | 0.29 | 2.22 | 1.62 | 0.29 | 0.10 | 0.50 | 2.36 | 1.15 | 3.88 | 1.77 | 0.94 | 2.90 |
| S_97.5_2 | -20.24 | -43.23 | 6.77 | 86.68 | -11.74 | 0.10 | 0.36 | 0.21 | 0.10 | 0.05 | 0.15 | 0.40 | 0.23 | 0.62 | 0.27 | 0.12 | 0.44 |
| S_97.5_3 | -9.40 | -27.83 | -4.60 | 28.08 | -14.85 | 0.19 | 0.46 | 0.36 | 0.24 | 0.07 | 0.55 | 0.50 | 0.24 | 0.79 | 0.44 | 0.17 | 0.91 |
| S_97.5_4 | -14.70 | -35.60 | 17.90 | 49.85 | -14.09 | 0.01 | 0.43 | 0.64 | 0.01 | 0.00 | 0.04 | 0.45 | 0.24 | 0.67 | 0.68 | 0.37 | 1.04 |
| S_97.5_6 | -6.52 | -7.17 | -5.87 | 19.53 | -12.28 | 0.30 | 0.26 | 0.33 | 0.35 | 0.16 | 0.56 | 0.31 | 0.14 | 0.50 | 0.39 | 0.18 | 0.62 |
| S_97.5_7 | -23.77 | -23.77 | -23.77 | 14.43 | -23.15 | 0.04 | 0.04 | 0.04 | 0.05 | 0.02 | 0.09 | 0.05 | 0.02 | 0.09 | 0.05 | 0.02 | 0.09 |
| S_97.5_8 | -2.70 | -3.07 | -2.33 | 15.72 | -11.31 | 0.55 | 0.52 | 0.57 | 0.54 | 0.27 | 0.88 | 0.52 | 0.26 | 0.85 | 0.56 | 0.28 | 0.92 |
| S_97.5_9 | -35.20 | -35.20 | -35.20 | 25.34 | -15.47 | 0.78 | 0.78 | 0.78 | 0.87 | 0.42 | 1.43 | 0.87 | 0.42 | 1.43 | 0.87 | 0.42 | 1.43 |
| S_97.5_10 | -23.77 | -23.77 | -23.77 | 34.25 | -20.61 | 0.09 | 0.09 | 0.09 | 0.10 | 0.05 | 0.17 | 0.10 | 0.05 | 0.17 | 0.10 | 0.05 | 0.17 |
| S_97.5_12 | -3.73 | -3.73 | -3.73 | 63.78 | -13.80 | 0.16 | 0.16 | 0.16 | 0.17 | 0.07 | 0.29 | 0.17 | 0.07 | 0.29 | 0.17 | 0.07 | 0.29 |
| S_97.5_21 | -23.77 | -23.77 | -23.77 | 18.27 | -22.82 | 0.05 | 0.05 | 0.05 | 0.06 | 0.03 | 0.10 | 0.06 | 0.03 | 0.10 | 0.06 | 0.03 | 0.10 |
| S_97.5_22 | -23.77 | -23.77 | -23.77 | 14.43 | -23.15 | 0.04 | 0.04 | 0.04 | 0.05 | 0.03 | 0.10 | 0.05 | 0.03 | 0.10 | 0.05 | 0.03 | 0.10 |
